# Influences of early diagnostic suggestions on clinical reasoning

**DOI:** 10.1186/s41235-022-00453-y

**Published:** 2022-12-15

**Authors:** Ploutarchos Kourtidis, Martine Nurek, Brendan Delaney, Olga Kostopoulou

**Affiliations:** Imperial College London, London, England UK

**Keywords:** Diagnosis, Confidence, Information search, Information evaluation, Resistance to change, Decision aids, Family medicine, Family practice, Clinical reasoning

## Abstract

Previous research has highlighted the importance of physicians’ early hypotheses for their subsequent diagnostic decisions. It has also been shown that diagnostic accuracy improves when physicians are presented with a list of diagnostic suggestions to consider at the start of the clinical encounter. The psychological mechanisms underlying this improvement in accuracy are hypothesised. It is possible that the provision of diagnostic suggestions disrupts physicians’ intuitive thinking and reduces their certainty in their initial diagnostic hypotheses. This may encourage them to seek more information before reaching a diagnostic conclusion, evaluate this information more objectively, and be more open to changing their initial hypotheses. Three online experiments explored the effects of early diagnostic suggestions, provided by a hypothetical decision aid, on different aspects of the diagnostic reasoning process. Family physicians assessed up to two patient scenarios with and without suggestions. We measured effects on certainty about the initial diagnosis, information search and evaluation, and frequency of diagnostic changes. We did not find a clear and consistent effect of suggestions and detected mainly non-significant trends, some in the expected direction. We also detected a potential biasing effect: when the most likely diagnosis was included in the list of suggestions (vs. not included), physicians who gave that diagnosis initially, tended to request less information, evaluate it as more supportive of their diagnosis, become more certain about it, and change it less frequently when encountering new but ambiguous information; in other words, they seemed to validate rather than question their initial hypothesis. We conclude that further research using different methodologies and more realistic experimental situations is required to uncover both the beneficial and biasing effects of early diagnostic suggestions.

**Supplementary Information:**

The online version contains supplementary material available at 10.1186/s41235-022-00453-y.

## Significance statement

Diagnostic errors can lead to patient harm and have been identified as a global priority by the World Health Organization. Suggesting to physicians diagnoses to consider before they start testing their own hypotheses has been found to increase accuracy in previous studies that used both online, rich clinical scenarios and simulated consultations with actors-as-patients. We explored hypothesised mechanisms of this phenomenon. We did not measure a clear and consistent effect of early diagnostic suggestions on physicians’ reasoning. In contrast, we measured a strong and consistent effect of confidence: when confidence about the initial diagnosis was high, it was followed by less extensive information search, more biased evaluations, fewer diagnostic changes, and fewer differential diagnoses. Thus, interventions that succeed in curbing physicians’ confidence in their initial diagnostic hypotheses may result in improved reasoning and greater accuracy. Furthermore, we raise the possibility that diagnostic suggestions may have an undesirable effect: when physicians see their own hypothesis amongst the suggestions, they may become more confident and more biased, validating rather than questioning their hypotheses.

## Introduction

Diagnosis is a core task in health care. It is particularly important in primary care, where patients present with new medical problems that need to be managed appropriately, without being indiscriminately subjected to invasive or expensive investigations (Singh et al., 2017). Failure to provide a timely and accurate diagnosis can have serious consequences for patients. Furthermore, it has been suggested that most people will experience at least one diagnostic error in their lifetime (IoM, 2015). It is therefore important to find ways of supporting medical diagnosis, as it is the task that leads to the greatest potential for serious medical error (Bhasale, 1998; Fisseni et al., 2008; Kostopoulou, 2006; Kostopoulou et al., 2008).

Medical diagnosis can be supported in different ways, including training, national guidelines, checklists, and decision aids. Decision aids are usually computerised tools, algorithms or online platforms that provide advice to support decision-making (Berner et al., 1999; Short, Frischer, & Bashford, 2003). In health care, this advice could range from risk calculation and procedural guidance to diagnostic suggestions and treatment plans.

Previous research has highlighted the importance of physicians’ initial hypotheses for their subsequent diagnostic judgements. For example, there is evidence that family physicians may miss cancers, if they do not consider them early on in the diagnostic process (Kostopoulou et al., 2017a). Thus, interventions to improve diagnosis could be more effective if they are employed early, before physicians start testing their own hypotheses. Based on this principle, Kostopoulou and colleagues (2015a, 2015b, 2017b) developed a computerised decision support tool, which provides diagnostic suggestions at the start of the consultation based on the patient’s demographics, risk factors and principal symptom. Two early studies evaluated the principle of early suggestions in two different countries (UK and Greece), with GPs diagnosing information-rich clinical scenarios online, where they could request information at will. A third study integrated the principle of early suggestions into a computerised diagnostic support tool and evaluated it in a high-fidelity simulation, where family physicians consulted with actors-as-patients (“standardised patients”). In all three studies, this type of decision support improved physicians’ diagnostic accuracy, without significantly increasing consultation time or number of investigations ordered (Kostopoulou et al., 2015a, 2015b, 2017a).

The mechanism by which early diagnostic suggestions impact clinical reasoning and improve diagnostic accuracy has not yet been explored. This is a crucial next step in the development of diagnostic aids. We need to understand why they are effective, i.e. how they influence physicians’ thinking, so that we can streamline and optimise them for clinical use.

Elstein, Shulman and Sprafka’s seminal studies found that physicians generate one or very few diagnostic hypotheses early in the consultation (i.e. within the first few seconds), based on minimal information (Elstein et al., 1978). Furthermore, the Hypothesis Generation (HyGene) model, a computational memory model, suggests that only a small number of hypotheses can be held in working memory due to memory constraints, and that these will guide the subsequent elicitation and interpretation of information (Thomas et al., 2008, 2014). These early hypotheses can, however, compromise the diagnostic process by crowding out other valid hypotheses, and exerting a disproportionate influence on what information is elicited and how it is interpreted (Brownstein, 2003). Thus, physicians may elicit information that is likely to confirm their focal hypothesis and/or interpret non-diagnostic information as supportive of that hypothesis. Even diagnostic information can be made to fit a coherent narrative that has developed during a consultation, as physicians, and perhaps even patients, search for cognitive consistency (Kostopoulou et al., 2009, 2012; Russo et al., 2008).

Coherent narratives induce greater confidence in judgement. “Confidence is a feeling, which reflects the coherence of the information and the cognitive ease of processing it” (Kahneman, 2011, p. 212). Thompson and colleagues (2011, 2013) suggested that people’s first intuitive judgements are accompanied by subjective confidence, a feeling that they are right. This can make people less open to alternative interpretations, less likely to seek additional information and re-evaluate their initial judgement and less likely to change it when appropriate (Desender et al., 2018; Thompson et al., 2011, 2013). These “feelings of rightness” about an initial intuitive judgement can determine whether, and to what extent, analytical reasoning will be activated.

Applying this to the clinical encounter, the first hypothesis that comes quickly to a physician’s mind will be accompanied by some degree of certainty, experienced as a feeling of rightness. If certainty is high, it could bias the subsequent diagnostic process and outcome. Indeed, overconfidence has been linked to diagnostic error (Berner & Graber, 2008; Friedman et al., 2005; Meyer et al., 2013). Berner and Graber (2008) suggest that physicians may develop an “illusion of validity”, which makes them overestimate the accuracy of their judgements (Einhorn & Hogarth, 1978).^1^ As a result, physicians often anchor on their initial diagnostic hypotheses and become less likely to seek advice or consider other possibilities (Arkes, 2013; Dreiseitl & Binder, 2005). When they do, they may selectively seek information that supports their hypotheses, (Dani, Bowen-Carpenter, & McGown, 2019; Mendel et al., 2011), and/or distort this information in favour their hypotheses (Kostopoulou et al., 2009, 2012; Leblanc et al., 2001, 2002; Nurek et al., 2014). Furthermore, studies have found physicians not to be well calibrated, i.e. their confidence did not match their accuracy (Dawson et al., 1993; Friedman et al., 2005; Meyer et al., 2013).

Presenting physicians with diagnostic alternatives early on could reduce unwarranted certainty by reminding them of other possibilities that they should consider. “Unpacking” hypotheses, i.e. presenting specific hypotheses in place of an “other” category, has been found to have a debiasing effect on diagnostic judgements and to reduce probability estimates attached to the focal hypothesis (Redelmeier et al., 1995). Furthermore, when physicians are presented with other possibilities, they may be more willing to seek further information before reaching a diagnostic conclusion, more cautious when they evaluate non-diagnostic information, and more likely to reconsider their initial diagnostic hypothesis.

Larrick categorised debiasing strategies into motivational, technological, and cognitive (Larrick, 2004). Motivational strategies try to leverage incentives, social norms, and accountability to improve decision-making and are related to the so-called choice architecture and nudging techniques (Dolan et al., 2012; Michie et al., 2011; Thaler & Sunstein, 2009). Technological strategies aim to improve decision-making through the use of algorithms and tools, such as decision analysis and computerised decision aids (Bhandari et al., 2008; Huang et al., 2012; Raiffa, 1968). Finally, cognitive strategies include training in logical rules, statistical reasoning and awareness of one’s own biases (Gigerenzer, 2015; Nisbett, 1993).

A well-known example of a cognitive debiasing strategy that encourages analytical reasoning is “consider-the-opposite”, a technique that directs attention towards disconfirming information and facilitates consideration of alternative hypotheses (Hirt & Markman, 1995; van Brussel et al., 2020). Along with decision aids, consider-the-opposite has been found to be one of the most effective debiasing strategies in health-related judgements (Ludolph & Schulz, 2017). For example, generating arguments that contradict one’s own hypotheses or favour alternative hypotheses has been found to reduce overconfidence (Haran, Moore, & Morewedge, 2010; Hirt & Markman, 1995; Koriat et al., 1980; McKenzie, 1997), anchoring (Mussweiler et al., 2000), confirmation bias (van Brussel et al., 2020), and hindsight bias (Arkes et al., 1988). Although it is classified as a cognitive debiasing strategy, research has shown that consider-the-opposite can be implemented through technological strategies as well, such as decision aids that provide alternative hypotheses for decision-makers to consider (Bhandari et al., 2008; Dreiseitl & Binder, 2005; Harada et al., 2021; Huang et al., 2012; Sibbald et al., 2021).

Based on these findings, we hypothesised that early provision of diagnostic suggestions would reduce certainty about an initial diagnostic hypothesis and as a consequence, lead to more extensive information search, more balanced appraisal of information, and more frequent diagnostic changes. To test these hypotheses, we conducted three online experiments where UK family physicians assessed hypothetical patient scenarios and either received a list of diagnostic suggestions or received no such help. Specifically, in Experiment 1, we tested the effects of early diagnostic suggestions on initial certainty and diagnostic change. In Experiment 2, we tested the effects of the suggestions on information search and information evaluation. In Experiment 3, we investigated these effects, after some modification on the list of suggestions.

In all the experiments, we accounted for Actively Open-minded Thinking (AOT). AOT refers to a thinking style that involves adopting various perspectives and considering arguments that oppose one’s own beliefs (Baron, 2019), seeking more information and considering alternatives (Baron, 2008; Haran, Ritov, & Mellers, 2013). Baron (2006) describes AOT as “good thinking”. It is a model of rational thinking that has been termed Active Open-mindedness because it is a) “open” to alternative explanations that oppose an initial judgement and b) “active” in searching for evidence to disconfirm pre-established beliefs (Baron, 2006, 2019). Baron further suggested that AOT is a way to prevent various biases to occur, including overconfidence and confirmation bias. To measure AOT, a scale was initially developed by Stanovich and West (1997). In our research, we used a shorter and more recent version of the scale developed by Baron (2019). It consists of eleven statements that measure how people evaluate information and form their beliefs (see Procedure).

## Experiment 1

In this within-participants experiment, we explored a potential mechanism by which early diagnostic suggestions might impact reasoning. Specifically, we tested whether diagnostic suggestions reduce physicians’ certainty about their initial diagnostic hypothesis, resulting in more frequent diagnostic changes when physicians encounter new information that is not entirely consistent with the initial hypothesis.

### Method

#### Participants and sample size

We powered the study to detect differences in diagnostic certainty between control and experimental conditions in a multiple linear regression. Using the G*power software (v. 3.1.9.4), we estimated that 392 responses would be needed to detect a small effect (Cohen’s *f*^2^ = 0.05) with 80% power and alpha of 0.05. To account for data clustering (each physician responding to two scenarios), we adjusted this number by the Design Effect (DE) (Barratt, Kirwan, & Shantikumar, 2018). This is calculated using the formula DE = 1 + (n–1)*ICC, where n is the cluster size (the two scenarios), and ICC is the intra-class correlation. The ICC of the original study was 0.05 (Kostopoulou et al., 2015b). Thus, DE = 1.05. We adjusted the number of participants required by multiplying the 392 required responses with the DE and dividing by the cluster size: (392*1.05)/2 = 205.8. Thus, we estimated that we needed to recruit 206 physicians.

We recruited fully qualified family physicians and trainees in family medicine, currently practising in England, using a database of family physicians who had participated in previous studies by the research group. We offered them a £10 Amazon voucher for their participation.

#### Materials

We used two patient scenarios, initially developed by Kostopoulou and colleagues (2017a) and adapted for the purposes of this experiment. One scenario depicted a patient presenting with chest pain, the other a patient presenting with breathlessness. For each scenario, we used the list of diagnostic suggestions prepared by Kostopoulou and colleagues (2017a): 18 diagnoses for one scenario and 23 for the other. The full scenarios with their lists of diagnostic suggestions are presented in the Additional file 1: S1.

#### Procedure

The experiment was conducted online and was administered using the Qualtrics platform. Participating physicians were sent an invitation e-mail that contained a brief description of the study and a hyperlink via which they could access the study website. Upon accessing the study, physicians read the information sheet and provided their consent. Subsequently, they were asked to indicate their gender (male or female), their professional status (fully qualified family physician or trainee). Fully qualified physicians were asked to provide their year of training completion. They were then presented with two patient scenarios in a random order. Only in one of these scenarios did participants receive diagnostic suggestions (the “Aided” condition). The provision of diagnostic suggestions was counterbalanced: half of the physicians received the suggestions in the first scenario and the other half in the second scenario. The Qualtrics randomiser ensured that each scenario was presented with and without diagnostic suggestions an equal number of times. Both scenarios were presented in two steps: at step one, physicians saw a brief patient description suggestive of a specific diagnosis. The description contained patient demographics, risk factors and the presenting problem. Participants were then asked to provide their initial diagnosis in a text box and indicate how certain they were on a visual analogue scale (VAS) ranging from 0 (Not at all certain) to 10 (Absolutely certain). At this stage, if the scenario was in the Aided condition, participants were instructed to read a list of diagnostic suggestions. They were told: “A decision aid trialled at your practice makes these diagnostic suggestions about the patient (in alphabetical order)”. To ensure that participants read the list, we used a timer that prevented physicians from progressing until 10 s had passed. At step two, participants were presented with additional information about the patient, including physical examination results. This information was somewhat ambiguous; it was consistent with the diagnosis suggested by the initial information but could also suggest other diagnoses. Participants were then asked to update their certainty about their initial diagnosis and provide their final diagnosis and certainty about the final diagnosis. Finally, they were asked to indicate whether they would order investigations and, if so, to specify which ones (in free text). After completing the two patient scenarios, participants completed the AOT scale (Additional file 1: S2). For each AOT statement, they indicated their agreement on a 5-point scale, ranging from “Completely disagree” to “Completely agree”.

#### Analyses

We computed change in initial certainty by subtracting the second measurement of initial certainty (elicited at step two) from the first measurement (elicited at step one). Change in diagnosis was a dichotomous variable (Yes/No) indicating whether the initial and final diagnoses differed. To determine this, we first standardised and classified diagnoses into diagnostic categories, under the guidance of the clinician co-author (BD), an experienced family physician, who was blinded to the experimental condition. For instance, angina, coronary artery disease, and ischaemic heart disease were classified as heart disease (see Additional file 1: S3). We then followed two criteria to determine whether a diagnostic change had occurred:

1. In case of a single initial diagnosis, any change in diagnosis, including a switch to a different diagnosis or an addition of a new diagnosis, counted as a change.
2. In case of multiple initial diagnoses, any change in the diagnostic set, such as the addition or removal of diagnoses, counted as a change. Number of investigations was a count variable indicating the number of different tests that physicians ordered in each scenario.

We regressed change in certainty, change in diagnosis, and number of investigations on condition (Aided vs. Control) in multilevel regression models with a random intercept by physician, controlling for initial certainty and AOT score. In a separate model, we added a variable indicating whether the suggestions were provided in the scenario seen first or second (Condition order). We used linear regression for continuous measures, logistic regression for dichotomous measures, and Poisson regression for the count variable. Table 1 presents a summary of the results across the two conditions.

**Table 1:** Descriptive statistics by condition

Outcome variable	Control condition	Aided condition
Mean initial certainty T1 (SD)	5.68 (1.62)	5.91 (1.53)
Mean initial certainty T2 (SD)	4.96 (2.17)	4.92 (2.12)
Mean change in initial certainty [T2-T1] (SD)	− 0.72 (1.63)	− 0.99 (1.72)
% change of initial diagnosis	46.90%	50.00%
Mean certainty for final diagnosis (SD)	5.62 (1.75)	5.71 (1.66)
Mean investigations (SD)	3.15 (2.49)	3.18 (2.41)

Change in initial certainty refers to the difference between the second (T2) and first (T1) measurements of initial certainty; on average, values were negative, indicating a reduction in certainty after participants saw the additional patient information at step 2

### Results

We recruited 217 family physicians. Twenty-one did not provide complete responses and two did not provide analysable diagnoses (e.g. “uncertain”); 23 physicians were thus excluded from the analyses. We analysed the responses of the remaining 194 physicians. There were 101 males (52%) and two were trainees. The sample’s average experience was 12.63 years in family medicine (SD 9.10), ranging from 0 to 41 years (median 10 years). Participation lasted on average 10 min and 7 s (range 3 to 34 min, median 9 min). Table 1 presents descriptive statistics for the Control and Aided conditions separately.

#### Change in initial certainty

Across conditions, certainty about the initial diagnosis dropped by an average of 0.85 units on the 0–10 scale. As expected, this reduction was greater in the Aided condition vs. Control (means − 0.99 vs. − 0.72); however, it was not significant (*b* =  − 0.239, [− 0.57, 0.09], *p* = 0.154). The higher the initial certainty, the less it dropped after additional information was provided (*b* =  − 0.149, [− 0.25, − 0.04], *p* = 0.007). When the suggestions were provided in the scenario seen first (vs. second), there was a greater reduction in certainty overall (means − 1.09 vs. − 0.63, *b* =  − 0.42 [− 0.76, − 0.09], *p* = 0.013), suggesting a possible spillover effect (from Aided to Control condition). We detected no association with the AOT score (*b* =  − 0.294, [− 0.82, 0.23], *p* = 0.272). The regression table is available in Additional file 1: S4.

**Fig. 1:**
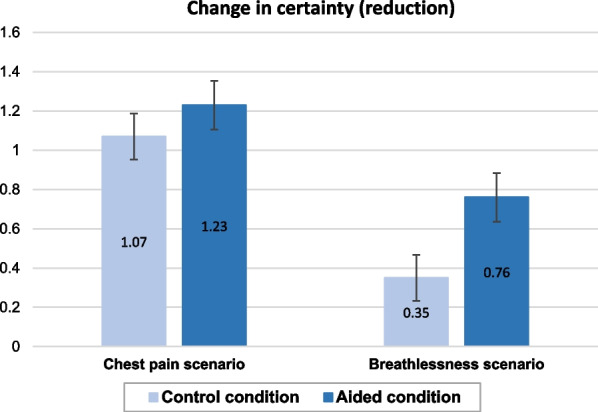
Mean change in certainty by condition and scenario

Figure 1 shows the mean change in certainty by Condition and scenario, where the bar height indicates the extent of change. We can see that in the breathlessness scenario, there was a greater difference in the extent of change between the two conditions than in the chest pain scenario. Indeed, subgroup analyses found that in the breathlessness scenario, initial certainty dropped significantly more in the Aided condition than Control (*b* =  − 0.41, [− 0.73, − 0.09], *p* = 0.013) but this difference was not significant in the chest pain scenario (*b* =  − 0.16, [− 0.74, 0.42], *p* = 0.583).

#### Change of initial diagnosis

Physicians changed their initial diagnosis 48.45% of the time. Results were according to expectations and consistent with the results on change in certainty. Specifically, diagnostic changes were more frequent when suggestions were provided (50% vs. 46.90%), but not significantly so (OR = 1.196, [0.79, 1.80], *p* = 0.389). Initial certainty was negatively associated with change in diagnosis (OR = 0.799, [0.70, 0.91], *p* = 0.001). When suggestions were provided in the scenario seen first (vs. second), the initial diagnosis changed more frequent (OR = 1.637, [1.08, 2.47], *p* = 0.019), again suggesting a spillover effect. There was no association with the AOT score (OR = 1.339, [0.71, 2.53], *p* = 0.367). The regression tables are available in Additional file 1: S4.

**Fig. 2:**
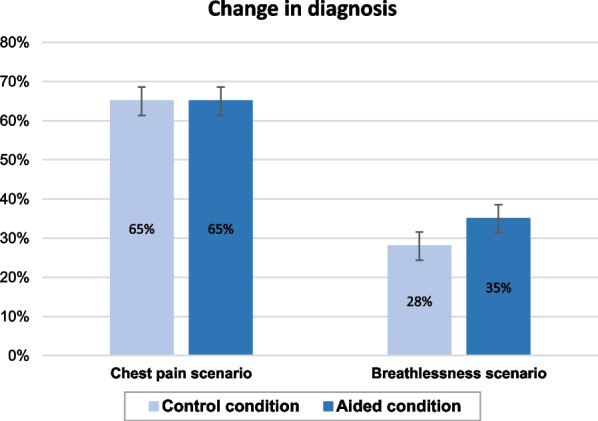
Frequency of diagnostic changes by condition and scenario

Figure 2 shows that in the chest pain scenario, diagnoses changed equally frequently in both conditions, whereas in the breathlessness scenario, there were more changes in the Aided condition, consistent with the greater reduction in certainty seen above. However, subgroup analyses did not detect a significant difference in the frequency of diagnostic changes between conditions in the scenario (OR = 1.38, [0.74, 2.54], *p* = 0.305).

#### Investigations

There was no significant difference between conditions in the number of investigations ordered (Control: 3.15 vs. Aided 3.18, IRR = 0.993, [0.89, 1.11], *p* = 0.907). The order in which suggestions were presented—in the scenario seen first vs. second, was not associated with number of investigations (IRR = 1.00, [0.97, 1.04], *p* = 0.714). We detected a positive association between AOT score and number of investigations (IRR = 1.47, [1.23, 1.75], *p* < 0.001). The regression table is presented in Additional file 1: S4.

### Discussion

We did not find that early suggestions significantly and consistently reduced diagnostic certainty or led to significantly more diagnostic changes compared to control. We did however find trends in the expected direction for both outcome variables, and a significant difference between conditions in one scenario. Active open-mindedness was associated with more investigations, as expected, but with no other outcome variable. Although the ambiguous additional information led to reductions in certainty across the board, when initial certainty was high, it was more resistant to change, and it was accompanied by fewer diagnostic changes.

There are several plausible reasons why diagnostic suggestions did not influence physicians’ responses significantly. Firstly, unexpected spillover effects from Aided to Control conditions may have compromised our ability to detect an effect. Secondly, it is possible that the patient scenarios did not have the anticipated result of inducing high initial certainty; this was, in fact, rather moderate, at around the midpoint of the scale. It is possible that physicians interpreted the initial information differently from what we had intended, given that physicians did not always provide the most likely initial diagnosis that each scenario suggested. For instance, in the chest pain scenario where the most likely diagnosis was musculoskeletal chest pain (Additional file 1: S3), physicians provided a different diagnosis 25% of the time (e.g. infection, pulmonary embolism, pericarditis). In the breathlessness scenario, responses were less variable (less than 10%). Importantly, we do not know to what extent physicians took the list of suggestions into account, since some commented that they found it lengthy, distracting, and confusing. Finally, it is possible that physicians approached the scenarios with an analytical mindset from the start, in the knowledge that they were being studied, which could reduce differences between conditions. We attempted to overcome some of these limitations in Experiment 2.

## Experiment 2

In this experiment, we used two new patient scenarios with strong signals aiming to induce higher initial certainty than in Experiment 1. Each scenario had two versions, one suggesting a serious and the other a less serious disease. This was to ensure that any effect of the diagnostic suggestions was not limited by the severity of the initial diagnosis. This also gave us the opportunity to investigate whether severity of the initial diagnosis was associated with subsequent information search and evaluation. We edited the list of diagnostic suggestions to maximise its impact, by removing the least likely diagnoses and merging any overlapping diagnoses. The number of diagnostic suggestions was therefore reduced and was equal in both scenarios (12 suggestions). Participants were able to request information about the patient by choosing from a list of clinical cues that were designed to provide non-diagnostic information. Thus, in addition to diagnostic certainty and diagnostic changes, we were able to measure information search, that is, the number of cues requested before a final diagnosis was given. We also measured information evaluation, by asking participants to rate the degree to which each requested cue supported their initial diagnosis. Diagnostic suggestions were always provided in the scenario seen last, to avoid spillover effects.

### Method

#### Participants and sample size

We powered the study to detect differences in cue ratings between Control and Aided conditions in a multiple linear regression. Using G*power software (v. 3.1.9.4), we estimated that to detect a small effect (*f*^2^ = 0.02)—smaller than in Experiment 1—with 80% power and alpha of 0.05, we would need 485 responses. To account for data clustering (each physician responding to two scenarios), we adjusted this number by the Design Effect (see Experiment 1) and estimated that we would need to recruit 254 physicians.

Participants were fully qualified family physicians and trainees in family medicine, currently practising in England. They were recruited through the National Institute for Health Research (NIHR) Clinical Research Network (CRN) of North-West London (www.local.nihr.ac.uk/lcrn/north-west-london) and were offered a £10 Amazon voucher as compensation for their time.

#### Materials

Two patient scenarios, previously developed by Kostopoulou and colleagues (2009, 2017a), were adapted for the purposes of this experiment. One scenario described a patient presenting with chest pain, the other a patient presenting with constipation. We constructed two versions of each scenario, one indicating a non-serious-disease and the other a serious-disease. In the non-serious-disease version, the patient’s initial information suggested a benign and common diagnosis (i.e. musculoskeletal chest pain in one scenario, irritable bowel syndrome in the other). The serious-disease version suggested a serious and less common diagnosis (i.e. angina in one scenario, colorectal cancer in the other). Each scenario also contained seven information items, identical in both versions, which participants could request. These were designed to be non-diagnostic. The scenarios with their respective lists of 12 diagnostic suggestions are presented in Additional file 1: S5.

#### Procedure

Potential participants were sent an invitation e-mail, containing a brief description of the experiment, as well as a hyperlink to access the study website. Upon accessing the study, physicians read the information sheet and provided their consent. They were then asked to indicate their gender (male, female, other), professional status (fully qualified family physician or trainee in family practice) and, if fully qualified, the year of qualification. Physicians were then presented with the patient scenarios in a random order. Scenario version (non-serious-disease vs. serious-disease) was assigned at random. The scenario seen first was presented without diagnostic suggestions (Control condition); the scenario seen second was presented with diagnostic suggestions (Aided condition). Each scenario was presented in two steps. Initially, participants read a short patient description including information such as age, body mass index (BMI), smoking and medical history, last consultation and presenting problem. They were then asked to provide their initial diagnosis in a text box and indicate how certain they were on a scale ranging from 0 (Not at all certain) to 10 (Absolutely certain). Subsequently, and in the Aided condition only, they were presented with a list of 12 diagnostic suggestions.

At step 2, all participants were given the opportunity to request up to seven additional items of information about the patient from a list of labelled clinical cues (e.g. general physical examination, family history of significant illness, pain intensity, previous episodes, other symptoms). Each cue contained neutral information with minimal diagnostic value (e.g. no family history of illness, normal resting electrocardiogram, no blood in stool). The list of cues was identical for both versions of a scenario. For each cue, participants were asked to rate how much the information supported their initial diagnosis, on a scale from 0 (No support) to 10 (Strong support). After each cue rating, they were asked to indicate whether they wished to provide a final diagnosis or request more cues. If they opted to request more cues, they were offered the remaining cues and asked to make a selection. If they indicated that they had settled on a diagnosis, or if they had requested all of the available cues, they were given the opportunity to review the patient case (i.e. the initial patient description plus any cues that they had requested) and then were asked to (1) update their certainty in the initial diagnosis; (2) provide their final diagnosis in free text; (3) rate their certainty in their final diagnosis; and (4) list the differential diagnoses that they were considering, if any. Finally, participants completed the AOT scale.

We expected that as physicians elicited more information by selecting from the list of cues, their certainty would increase rather than decrease, because the cues did not contradict the initial patient description; they were simply non-diagnostic, i.e. did not provide evidence in support of the steered diagnosis. We did however expect that in the Aided (vs. Control) condition, this increase in certainty would be smaller. Furthermore, in the Aided condition, we expected that more cues would be requested; they would be rated as less supportive of the physicians’ initial diagnosis; and initial diagnoses would change more frequently.

#### Analyses

Change in initial certainty, change in diagnosis, and AOT scores were computed as per Experiment 1. Number of cues requested was a count variable with possible values from one to seven, which was the maximum number of cues available to physicians. Perceived cue support (measured on a 0–10 scale) was averaged across elicited cues per physician. Number of differential diagnoses was a count variable corresponding to the number of alternative diagnoses that physicians recorded after providing their final diagnosis. Severity of initial diagnosis was coded as “1” for serious and “0” for non-serious diagnoses.

As in Experiment 1, we standardised and classified diagnoses into diagnostic categories (Additional file 1: S6). We also categorised participants’ initial diagnoses based on their severity as either serious or non-serious. We regressed the outcome variables (number of items requested, perceived cue support, change in certainty, change in diagnosis, number of differential diagnoses) on Condition (Aided vs. Control) in multilevel regressions with random intercept per participant, controlling for initial certainty, severity of initial diagnosis, and AOT score. We used linear regression for continuous measures, logistic regression for dichotomous measures and Poisson regression for the count variables.

### Results

**Fig. 3:**
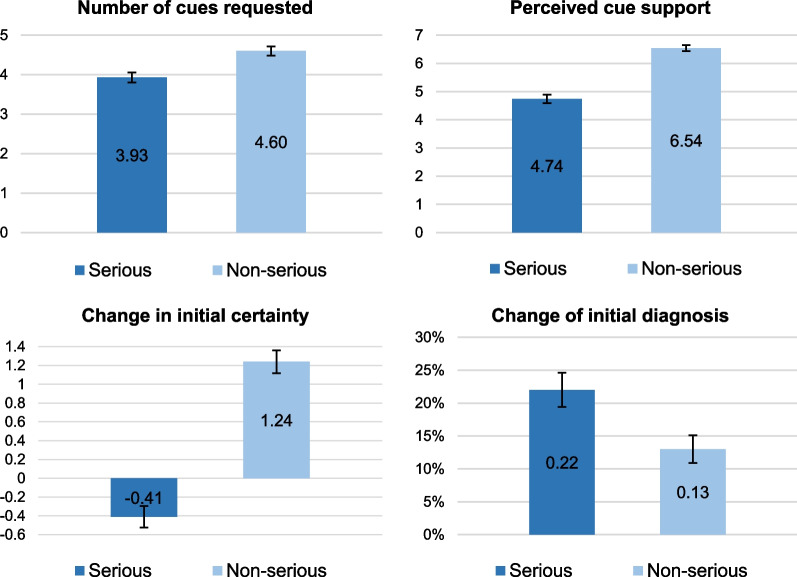
Results of the variables of interest by severity of initial diagnosis. Top left panel: mean number of cue requests; Top right panel: mean perceived cue support (scale); Bottom left panel: mean change in initial certainty (scale); bottom right panel: percentage change of initial diagnosis following cue requests. All the differences were significant

We recruited 273 physicians. Fifteen of them did not complete the task and another 10 provided non-analysable responses and were therefore excluded from the analyses. Of the remaining 248 participants, 115 were males (46.40%) and 223 were fully qualified family physicians (89.90%) with on average 10.44 years’ clinical experience post-qualification (SD 9.06, range 0 to 44 years, median 9 years). Participation lasted on average 16 min (range 3 to 33 min, median 13 min). Table 2 presents descriptive statistics for the Control and Aided conditions separately. Figure 3 presents a summary of results for the main variables of interest by severity of initial diagnosis. GPs provided the expected diagnosis 78.83% of the time (391/496, Table 3).

**Table 2:** Descriptive statistics by condition

Outcome variable	Control condition	Aided condition
Mean initial certainty T1 (SD)	6.11 (1.87)	6.11 (1.79)
Mean initial certainty T2 (SD)	6.50 (2.30)	6.53 (2.05)
Mean change in initial certainty [T2-T1] (SD)	0.40 (2.16)	0.42 (1.87)
Number of cues requested	4.16 (2.06)	4.37 (1.87)
Perceived cue support	5.46 (2.54)	5.82 (2.00)
% change of initial diagnosis	19.00%	15.00%
Mean certainty for final diagnosis (SD)	7.06 (1.58)	6.73 (1.80)
Mean differential diagnoses (SD)	1.32 (1.18)	1.25 (1.08)

Initial certainty T2 is the certainty in the initial diagnosis measured after participants requested and received additional cues and before they provided their final diagnosis. Change in initial certainty refers to the difference between the second and the first measurements of initial certainty and was on average positive indicating an increase in certainty

**Table 3:** Diagnoses by scenario version

Scenario version	Scenario severity	% accuracy	% serious diagnoses	% non-serious diagnoses
Musculoskeletal chest pain	Non serious	84.68% (105/124)	15.32% (19/124)	84.68% (105/124)
Irritable bowel syndrome	Non serious	70.97% (88/124)	20.16% (25/124)	79.84% (99/124)
Angina	Serious	97.58% (121/124)	97.58% (121/124)	2.42% (3/124)
Colorectal cancer	Serious	62.10% (77/124)	67.74% (84/124)	32.26% (40/124)

Scenario severity is the severity of the disease that the scenario version intended to depict (the expected diagnosis); “% accuracy” is the proportion of initial diagnoses that matched the expected diagnoses. “% serious/non-serious diagnoses” are proportions of responses that provided a serious/non-serious initial diagnosis

#### Cue requests

On average, participants requested 4.26 cues (SD 1.97). As expected, they requested more cues when they received diagnostic suggestions than when they did not (4.16 vs. 4.37), but this difference was not significant (IRR = 0.950, [0.87, 1.03], *p* = 0.235). There was a negative association with initial certainty (IRR = 0.918, [0.90, 0.94], p < 0.001); a positive association with AOT scores (IRR = 1.370, [1.22, 1.53], *p* < 0.001); and no association with severity of initial diagnosis (IRR = 1.055, [0.97, 1.15], *p* = 0.228). The regression table is presented in Additional file 1: S7.

#### Perceived cue support

Across scenarios and conditions, the average perceived cue support was 6.11 (SD 1.97) on the 0–10 scale. Contrary to our hypothesis, cue information was rated as more supportive of the initial diagnoses in the Aided (vs. Control) Condition (*b* = 0.367, [0.08, 0.66], *p* = 0.013). When physicians provided a non-serious (vs. serious) initial diagnosis, they perceived the cue information as more supportive of it (*b* =  − 2.05, [− 2.39, − 1.71], *p* < 0.001). There was a positive association between perceived cue support and initial certainty (*b* = 0.517, [0.42, 0.61], *p* < 0.001). No association with the AOT score was detected (*b* = 0.041, [− 0.43, 0.51], *p* = 0.864). The regression table is presented in Additional file 1: S7.

#### Change in initial certainty

After additional cues were obtained, we measured a small change in certainty about the initial diagnosis and no significant difference between conditions (*b* = 0.034, [− 0.27, 0.34], *p* = 0.829). The change was smaller for serious than non-serious initial diagnoses (*b* =  − 1.413, [− 1.74, -1.09], *p* < 0.001). In fact, certainty about serious initial diagnoses reduced on average (mean − 0.41, SD 1.80), while it increased on average for non-serious diagnoses (mean 1.24, SD 1.89). No association with AOT score was detected (*b* = 0.101, [− 0.30, 0.50], *p* = 0.618). The regression table is presented in Additional file 1: S7.

#### Change of initial diagnosis

Overall, participants changed their initial diagnosis 17.34% of the time. Contrary to expectations, changes were more frequent in the Control than the Aided condition (Table 2). However, the difference was not significant (OR = 0.816, [0.50, 1.33], *p* = 0.411). Serious initial diagnoses were more likely to change than non-serious ones (OR = 2.50, [1.50, 4.18], *p* < 0.001). Initial certainty was negatively associated with diagnostic change (OR = 0.680, [0.59, 0.78], *p* < 0.001). No association with the AOT score was detected (OR = 1.028, [0.56, 1.90], *p* = 0.929). The regression table is presented in Additional file 1: S7.

#### Differential diagnoses

On average, physicians recorded 1.28 differential diagnoses (SD 1.13, range 0 to 7 diagnoses, mode 1), with no significant difference between conditions (IRR = 1.062, [0.91, 1.23], *p* = 0.430). There was no association with the severity of initial diagnosis (IRR = 0.869, [0.74, 1.01], *p* = 0.074) and a positive association with the AOT score (IRR = 1.212, [1.00, 1.47], *p* = 0.049). Higher initial certainty was associated with fewer differential diagnoses (IRR = 0.951, [0.91, 0.99], *p* = 0.018).

### Discussion

Experiment 2 did not detect any effect of the diagnostic suggestions on the measures of interest. As expected, physicians requested more cues when they received diagnostic suggestions than when they did not, but the difference was not significant. In contrast to our hypothesis, they also rated cues as significantly more supportive of their initial diagnoses. Furthermore, they changed diagnosis less frequently than in the control condition, but this difference was not significant.

The severity of the initial diagnosis seemed an important factor in how information was evaluated. Certainty about non-serious initial diagnoses increased significantly, which was probably supported by a more biased cue evaluation than an initial serious diagnosis. Moreover, non-serious diagnoses changed less frequently than serious ones. Given that non-serious diagnoses are common in primary care and that the cue information had minimal diagnostic value, it is not a surprise that physicians maintained them, though we had expected more changes in the Aided condition.

As in Experiment 1, the main driver of behaviour was initial diagnostic certainty: higher certainty was associated with fewer cue requests, more biased cue evaluation (i.e. higher perceived cue support), fewer diagnostic changes and fewer diagnostic alternatives offered. A change in physicians’ certainty about their initial diagnostic hypotheses could, therefore, have a ripple effect on the diagnostic process; the Aided condition failed to produce such a change.

We cannot attribute this failure to idiosyncrasies of the scenarios, since by and large they worked as expected. In an attempt to address the limitations of Experiment 1, specifically, to reduce the variability in initial diagnostic hypotheses and induce higher initial certainty—we included stronger signals in the initial patient description. Indeed, most participants gave the initial diagnoses that we expected and did not change them, even though initial certainty was still moderate. Furthermore, we improved the list of diagnostic suggestions, removing unlikely and superfluous diagnoses, to increase its perceived relevance, usefulness and impact. We precluded potential spillover effects by ensuring that the Aided condition always appeared last. Finally, we introduced new measures for information search and evaluation to increase the chance of detecting an effect of diagnostic suggestions. Still, we observed no effect.

It is possible that the list of suggested diagnoses did not serve as intended, i.e. to create doubt in physicians about their initial diagnosis by presenting them with diagnostic alternatives. Instead, it may have served as a means for them to validate their own initial hypotheses. In most cases, the physicians’ initial diagnoses were included in the list of suggestions. Seeing their own diagnosis on the list may have reassured them that it was correct. As Ridderikhoff & van Herk pessimistically put it, “The look at the ddx^2^ list seems to serve only one purpose: the verification of the diagnostic assumption” (p. 98) (Ridderikhoff & van Herk, 1999). We tested this hypothesis in Experiment 3.

## Experiment 3

The present experiment tested the possibility that physicians used the suggestions to validate their own initial diagnosis rather than to question it. We speculated that the inclusion of the physicians’ own diagnoses in the list of suggestions could have been a confirmatory and reassuring sign that they were following the correct diagnostic path. Thus, we removed the most likely diagnosis from the list of suggested diagnoses, as this was the diagnosis that most participants initially gave and asked them to “also consider the following possible diagnoses for the patient”. We recruited an entirely new sample of family physicians for Experiment 3. They saw only one scenario, always with diagnostic suggestions. All other procedural aspects remained the same as in Experiment 2. We compared the responses of the new sample with the responses provided in the Control and Aided conditions of Experiment 2. We expected that removing the most likely diagnosis from the list of suggestions would impact diagnostic certainty, causing either a reduction or a smaller increase than in Experiment 2. Associations with severity of initial diagnoses and AOT scores were also explored.

### Method

#### Participants and sample size

Participants were qualified family physicians and trainees in family medicine, currently practising in England. We recruited the same number of participants as in Experiment 2 (*N* = 248). Participants were recruited through the North-West London Clinical Research Network and were offered a £10 Amazon voucher for their participation.

#### Materials

We used the same materials as in Experiment 2. The only difference was that the list of diagnostic suggestions did not include the most likely diagnosis for each scenario version. Specifically, in the serious-diagnosis version of the chest pain scenario, angina was removed from the list; in the non-serious-diagnosis version, musculoskeletal chest pain was removed from the list. For the constipation scenario, colorectal cancer was removed from the list in the serious-diagnosis version and irritable bowel syndrome was removed from the list in the non-serious-diagnosis version.

#### Procedure

Participants were randomly assigned to view only one scenario version from the four possible, always with diagnostic suggestions. Each scenario version was seen an approximately equal number of times. In all other respects, the procedure was identical to the Aided condition of Experiment 2.

#### Analyses

The data from Experiments 2 and 3 formed three conditions:

1. Control condition of Experiment 2 (scenarios seen without diagnostic suggestions).
2. Aided condition of Experiment 2 (“Aided 1”) (scenarios seen with diagnostic suggestions, including the most likely diagnosis).
3. Aided condition of Experiment 3 (“Aided 2”) (scenarios seen with diagnostic suggestions, excluding the most likely diagnosis).

Experiment 2 had followed a within-participants design, where physicians saw two scenarios, one with and the other without suggestions. Therefore, Control and Aided 1 responses were not independent. Aided 2 responses were elicited from an entirely different sample of physicians who saw one of the two scenarios used in Experiment 2. For this reason, we conducted two separate comparisons: Control vs. Aided 2 and Aided 1 vs. Aided 2. As in Experiments 1 and 2, we excluded non-analysable responses. We also excluded from all conditions (Control, Aided 1, Aided 2) responses where the physician did not provide the most likely diagnosis as their initial diagnosis. This was to ensure that physicians were not able to validate their diagnosis if this was included in the list of suggestions.

As in Experiment 2, we simplified physicians’ diagnoses (Additional file 1: S6) and measured the same variables (initial certainty, number of cues requested, perceived cue support, change in certainty, change in diagnosis, certainty in final diagnosis, and number of differential diagnoses) using the same scales. We run the same analyses using linear, logistic and Poisson regression models but this time as simple regression models (not multilevel) as each comparison (Aided 2 vs. Control and Aided 2 vs. Aided 1) used one scenario per physician.

### Results

**Fig. 4:**
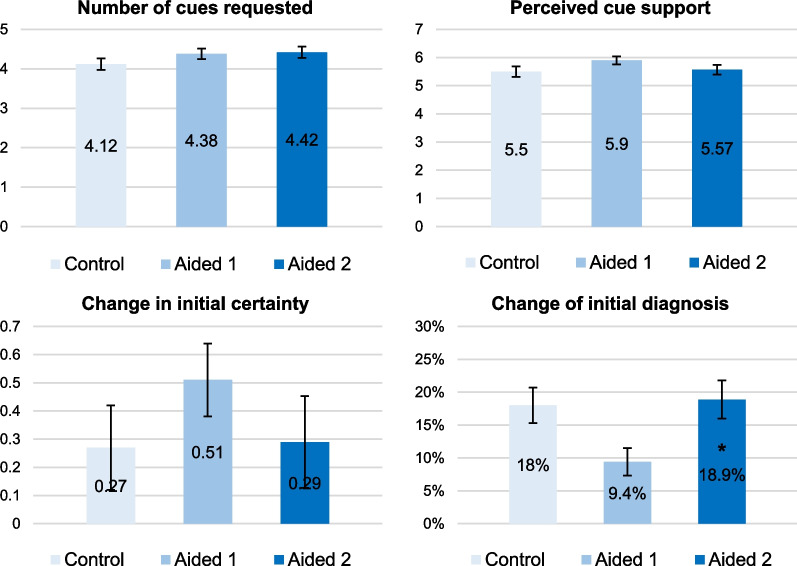
Results of the variables of interest by condition. Top left panel: mean number of cue requests; Top right panel: mean perceived cue support (scale); Bottom left panel: mean change in initial certainty (scale); bottom right panel: percentage change of initial diagnosis following cue requests. * denotes significance at the level of 0.05 between Aided 1 and Aided 2

We recruited 258 family physicians. Eight of them were excluded due to incomplete data and another two due to non-analysable responses. From the remaining 248 participants, 58 physicians (23.40%) did not provide the expected diagnoses and were also excluded. The final sample consisted of 190 participants: 74 males (38.90%) and 172 physicians fully qualified in family medicine (90.50%), with average experience of 10.33 years (SD 8.74, range 0–36 years, median 7 years). After we also excluded physicians’ responses that did not contain the expected diagnoses in Experiment 2, a total of 200 Control responses and 191 Aided 1 responses were included in the analyses. The participation lasted on average 13 min (range 3 to 33 min, median 10 min). Table 4 presents descriptive statistics by condition. Figure 4 presents a summary of results by condition for the main variables of interest.

**Table 4:** Descriptive statistics by condition

Outcome variable	Control	Aided 1	Aided 2
Mean initial certainty T1 (SD)	6.38 (1.77)	6.35 (1.66)	6.13 (1.76)
Mean initial certainty T2 (SD)	6.64 (2.27)	6.86 (1.84)	6.42 (2.47)
Mean change in initial certainty (T2-T1) (SD)	0.27 (2.12)	0.51 (1.78)	0.29 (2.24)
Mean cue requests (SD)	4.12 (2.10)	4.38 (1.85)	4.42 (1.96)
Mean perceived cue support (SD)	5.50 (2.60)	5.90 (1.99)	5.57 (2.39)
% change of initial diagnosis	18.00%	9.40%	18.90%
Mean certainty about the final diagnosis (SD)	7.19 (1.50)	6.97 (1.67)	6.95 (1.82)
Mean differential diagnoses (SD)	1.26 (1.15)	1.25 (1.07)	1.34 (1.11)

Mean change in initial certainty was positive in all conditions (increase)

#### Cue requests

On average, participants requested 4.30 cues per scenario (SD 1.98), with the most cue requests in Aided 2 condition. No significant differences between conditions were detected (Control vs. Aided 2: IRR = 0.959, [0.87, 1.06], *p* = 0.389 and Aided 1 vs. Aided 2: IRR = 1.015, [0.92, 1.12], *p* = 0.762). As in Experiment 2, initial certainty was negatively associated with cue requests in both comparisons (Control vs. Aided 2: IRR = 0.906, [0.88, 0.93], *p* < 0.001 and Aided 1 vs. Aided 2: IRR = 0.915, [0.89, 0.94], *p* < 0.001). Similarly, AOT scores were positively associated with cue requests, but this was significant only in one comparison (Control vs. Aided 2: IRR = 1.189, [1.04, 1.35], *p* = 0.008 and Aided 1 vs. Aided 2: IRR = 1.113, [0.97, 1.27], *p* = 0.115). There were no associations with severity of initial diagnosis (Control vs. Aided 2: IRR = 0.997, [0.90, 1.10], *p* = 0.957 and Aided 1 vs. Aided 2: IRR = 1.042, [0.95, 1.15], *p* = 0.400). The regression tables are presented in Additional file 1: S8.

#### Perceived cue support

Perceived cue support in the Control condition was highly variable between scenarios and within scenario versions (Additional file 1: S9). In the chest pain scenario, the mean ratings ranged from 4.94 (angina version) to 6.32 (musculoskeletal chest pain version), whereas in the constipation scenario, the ratings ranged from 3.16 (colorectal cancer version) to 7.29 (irritable bowel syndrome version), on the 0–10 scale. Average perceived cue support was 5.65 (SD 2.35), the highest being in Aided 1 condition. There were no significant differences between conditions (Control vs. Aided 2: *b* = 0.100, [− 0.10, 0.30], *p* = 0.335 and Aided 1 vs. Aided 2: *b* =  − 0.230, [− 0.60, 0.14], *p* = 0.226). As in Experiment 2, non-serious (vs. serious) initial diagnoses were accompanied by more biased evaluation of information for both comparisons (Control vs. Aided 2: *b* =  − 2.709, [− 3.13, − 2.29], *p* < 0.001 and Aided 1 vs. Aided 2: *b* =  − 2.074, [− 2.45, − 1.70], *p* < 0.001). As in Experiment 2, initial certainty was positively associated with cue evaluations, i.e. higher perceived cue support for both comparisons (Control vs. Aided 1: *b* = 0.461, [0.34, 0.58], *p* < 0.001 and Aided 1 vs. Aided 2: *b* = 0.452, [0.34, 0.56], *p* < 0.001). No associations with the AOT score were detected (Control vs. Aided 2: *b* = 0.189, [− 0.34, 0.72], *p* = 0.485 and Aided 1 vs. Aided 2: *b* = 0.403, [− 0.10, 0.904], *p* = 0.115). The regression tables are presented in Additional file 1: S8.

#### Change in initial certainty

Across scenarios and conditions, initial certainty increased by an average of 0.35 units on the 0–10 scale, with the highest increase in Aided 1 condition. No significant differences between conditions were detected (Control vs. Aided 2: *b* =  − 0.013, [− 0.20, 0.17], *p* = 0.889 and Aided 1 vs. Aided 2: *b* =  − 0.288, [− 0.65, 0.07], *p* = 0.119). Serious initial diagnoses were associated with smaller change in initial certainty than non-serious diagnoses across comparisons (Control vs. Aided 2: *b* =  − 1.868, [− 2.25, − 1.48], *p* < 0.001 and Aided 1 vs. Aided 2: *b* =  − 1.309, [− 1.68, − 0.94], *p* < 0.001). No associations with the AOT score were detected (Control vs. Aided 1: b = 0.139, [-0.35, 0.63], p = 0.574 and Aided 1 vs. Aided 2: *b* = 0.279, [− 0.21, 0.77], *p* = 0.262). The regression tables are presented in Additional file 1: S8.

#### Change of initial diagnosis

Physicians changed their diagnosis in 15.50% of the responses, with most changes in the Aided 2 condition. The difference between Aided 1 and Aided 2 was significant (OR = 2.143, [1.14, 4.03], *p* = 0.018), but the difference between Control and Aided 2 was not (Control vs. Aided 2: OR = 0.960, [0.73, 1.26], *p* = 0.769). As in Experiment 2, initial non-serious diagnoses changed less frequently in the Control condition than the Aided 2 condition (Control vs. Aided 2: OR = 2.779, [1.55, 4.97], *p* = 0.001); no significant difference was detected in the other comparison (Aided 1 vs. Aided 2: OR = 1.727, [0.93, 3.22], *p* = 0.085). As in both Experiments 1 and 2, initial certainty was negatively associated with diagnostic change (Control vs. Aided 2: OR = 0.623, [0.53, 0.73], *p* < 0.001 and Aided 1 vs. Aided 2: OR = 0.632, [0.53, 0.76], *p* < 0.001). No associations with the AOT score were detected (Control vs. Aided 2: OR = 0.990, [0.48, 2.03], *p* = 0.979 and Aided 1 vs. Aided 2: OR = 0.667, [0.28, 1.57], *p* = 0.354). The regression tables are presented in Additional file 1: S8.

#### Differential diagnoses

On average, physicians recorded 1.27 differential diagnoses (range 0 to 8 diagnoses, median 1), the most in the Aided 2 condition (mean 1.34, SD 1.11). There were no significant differences between conditions (Control vs. Aided 2: IRR = 0.969, [0.81, 1.15], *p* = 0.721 and Aided 1 vs. Aided 2: IRR = 0.924, [0.77, 1.10], *p* = 0.389). As in Experiment 2, initial certainty was negatively associated with the number of differential diagnoses (Control vs. Aided 2: IRR = 0.925, [0.88, 0.97], *p* = 0.001 and Aided 1 vs. Aided 2: IRR = 0.907, [0.86, 0.95], *p* < 0.001). Significantly more differential diagnoses were recorded when the initial diagnosis was serious in Aided 2 than Aided 1 (IRR = 0.805, [0.67, 0.96], *p* = 0.019); no such association was detected in the comparison between Control and Aided 2 (IRR = 0.892, [0.75, 1.07], *p* = 0.208). Higher AOT scores were associated with more differential diagnoses in Aided 2 than Control (Control vs. Aided 2: IRR = 1.405, [1.11, 1.78], *p* = 0.005); no such difference was detected in the other comparison (Aided 1 vs. Aided 2: IRR = 1.228, [0.96, 1.58], *p* = 0.108).

### Discussion

Overall, Experiment 3 produced results consistent with Experiment 2, detecting mostly trends rather than significant differences between conditions. As expected, physicians in the Aided 2 condition requested the most cues, changed their initial diagnosis most frequently, and recorded the most differential diagnoses. In fact, diagnostic changes were significantly more frequent when the most likely diagnosis was removed from the list of suggestions (Aided 2) than when it was included (Aided 1). However, no differences from Control were detected. We also measured the most biased cue evaluation (highest perceived cue support) and the largest increase in certainty when the most likely diagnosis was included in the list (Aided 1)—though differences from Aided 2 were not significant. Thus, our results highlight a potential peril of decision support: when physicians see their focal diagnosis in the list of suggestions (Aided 1), they may become closed- rather than open-minded, feel more certain, and fail to consider other diagnoses. Removing the most likely diagnosis from the list seemed to counteract the bias but did not provide additional improvement compared to Control.

As in Experiment 2, the variable that affected all variables of interest and always in the expected direction was initial certainty. Higher initial certainty was consistently accompanied by significantly higher perceived cue support, fewer cue requests, fewer diagnostic changes, and fewer differential diagnoses.

Perceived cue support was highly variable, which could have prevented between-condition differences to emerge. The baseline ratings of physicians’ perceived cue support in the Control condition varied widely both between and within scenarios (i.e. between versions of the same scenario, see Additional file 1: S9). For instance, physicians who saw the non-serious-diagnosis version of the chest pain scenario evaluated the cue information as more supportive of their initial diagnosis (mean 6.32 support for musculoskeletal) than those who saw the serious-diagnosis version (mean 4.94 support for angina). Likewise, in the constipation scenario, physicians found more support for the non-serious diagnosis (mean 7.29 support for irritable bowel syndrome) than the serious one (mean 3.16 support for cancer). This suggests that although the cues were designed to be neutral for both diagnoses (serious and non-serious), their perceived diagnosticity differed across the scenario versions. It is possible that the severity, as well as the familiarity of the diagnosis, had an impact on physicians’ evaluation of information, who found more support for common and less serious diagnoses than uncommon and serious ones.

As other studies have found, clinical cues are not evaluated independently and in isolation but within a developing, explanatory narrative of the patient problem (Kostopoulou et al., 2009, 2012). Even within a single scenario version, physicians perceived some cues to be more supportive of their diagnosis than others; for instance, in the cardiac version of the chest pain scenario, the self-treatment cue information had an average rating of 2.94, whereas the pain intensity cue information had an average rating of 6.05. Despite having been designed to be neutral, physicians did not always perceive cues as such and attached differential diagnostic value to them. This variability in the interpretation of the neutral cues depending on initial diagnosis and scenario version could have reduced the opportunity to find consistent differences between conditions.

## General discussion

This research consisted of three experiments testing the effects of early diagnostic suggestions on physicians’ reasoning. The experiments investigated different aspects of the diagnostic process including diagnostic certainty, information search, information evaluation and diagnostic changes when encountering new information. Using clinical scenarios presented online, we tested whether providing diagnostic suggestions early in the process can reduce the biasing effects of overconfidence. We did not detect significant differences from control; instead, we present some evidence that including the most likely diagnosis in the list of suggestions may operate as a validation rather than a debiasing tool. As expected, active open-mindedness was associated with more investigations, more cue requests, and more differential diagnoses.

Irrespective of the provision of diagnostic suggestions, it was initial certainty that was the main driver of behaviour: high certainty led to significantly fewer information requests (also see Meyer et al., 2013), more biased information evaluations, and fewer changes in diagnosis when encountering new information that either suggested additional possibilities or did not entirely fit with one’s leading hypothesis. The negative relationship between initial certainty and change in diagnosis was consistent across the three experiments. This finding is in line with previous research on Feelings of Rightness (i.e. subjective confidence in an initial judgement) and resistance to changing one’s mind (Thompson et al., 2013; Wang & Thompson, 2019). Previous research has also shown that confident initial judgments and first impressions are less likely to change in individual as well as in group decision-making (Kruglanski et al., 1993). Similar results have been found in a range of judgments, including consumer choices (Folke et al., 2017), syllogistic reasoning tasks (Shynkaruk & Thompson, 2006; Thompson et al., 2011, 2013; Wang & Thompson, 2019), medical diagnoses and treatment decisions (Dreiseitl & Binder, 2005; Jaimes et al., 2013; Krupat et al., 2017; Pandharipande et al., 2016), and moral dilemmas (Vega et al., 2020). The phenomenon is therefore not limited to lay people, but it is also applicable to experienced professionals. Our findings complement previous research and highlight the importance of the first (intuitive) judgments for the final decision (Ames et al., 2010; Hogarth & Einhorn, 1992; Kahneman, 2011; Kahneman & Tversky, 1974; Kostopoulou et al., 2017b; Stone, 1994).

There are several reasons that could explain why we did not detect any measurable and consistent impact of diagnostic suggestions on physicians’ behaviour. Firstly, the scenarios did not induce high diagnostic certainty. In all three experiments, certainty about the first diagnostic hypothesis was moderate. This could indicate that physicians were already sceptical about their initial diagnosis or not prepared to declare high confidence given the limited amount of information. If physicians were *not* especially certain about their initial diagnosis—and the list of suggestions aimed to reduce that initial certainty—then there may have been little for the list to “do”. More formally: moderate initial certainty could have weakened any debiasing effect of the diagnostic suggestions on physicians’ thinking.

Secondly, the conditions and task demands in the experiments may not reflect those of the original experiments where the phenomenon was first established (Kostopoulou et al., 2015a, 2015b; Kostopoulou et al., 2017a). In the original experiments, physicians were able to elicit information at will, either from actors or while on the phone with a researcher, who responded to their information requests by sending the answer to their screens. Thus, the interaction was rich and realistic, as if they were talking to a patient. This may have motivated them to take account of the list of suggestions more than in the present experiments.

In the original experiments, physicians could request substantially more information than in the present experiments where only seven items were available to them. Thus, they had more opportunity to test other hypotheses. In fact, an analysis of physicians’ written documentation of simulated consultations with actors, obtained by Kostopoulou et al., (2017a), found that in unaided consultations (Control), physicians recorded predominantly observations related to their final diagnosis, suggesting an almost exclusive focus on that diagnosis, while in the aided consultations, they also recorded other observations as they were exploring additional possibilities (Kostopoulou et al., 2021).

In the original experiments, physicians were not asked to provide explicit certainty ratings about their initial diagnosis, as was done in the present experiments. Asking people to state their confidence explicitly may make them unwilling to change it when encountering new information that does not confirm what they are thinking. Thus, explicit statements of confidence may have acted as an anchor for the subsequent confidence ratings. Across the three experiments, the average change in certainty was less than 1 unit on the 0–10 scale. The same may have occurred with the requirement to provide a single diagnostic hypothesis early on and with limited information, reducing physicians’ willingness to change diagnosis when more and ambiguous information was revealed.

The original experiments required substantial involvement by the participants but also offered them substantial rewards. For the experiments using computerised clinical scenarios (Kostopoulou et al., 2015a, 2015b) physicians were invited and remunerated for a 3-h involvement—they did 9 scenarios and could ask any question they wanted (they asked 19 questions on average). They were also offered individualised feedback, which they could use as evidence of continuous professional development. In the experiment with standardised patients, participants consulted with 12 actors over 2 different days and were videotaped. They were recompensed for their time. In summary, the original experiments were substantially longer, more realistic, offered significant benefits and rewards, and hence could secure serious participant involvement that the present experiments perhaps did not. Finally, in the original experiments, physicians diagnosed 9–12 different scenarios/standardised patients, whereas in the present experiments, they responded to 1–2 scenarios only. The large variability in responses between these scenarios may have prevented us from detecting an impact of the diagnostic suggestions.

Future studies could increase the realism of the diagnostic situation by having extensive patient information available to physicians, thus providing more opportunities for active engagement; and using scenarios that allow for a final diagnosis—which could improve participants’ motivation and engagement. The use of process tracing methodologies such as eye tracking could also reveal if and how physicians interact with the decision aid by measuring number and length of fixations (Schulte-Mecklenbeck et al., 2019).

A study from researchers independent from our group has found that early diagnostic suggestions improve diagnostic accuracy by enabling physicians to consider more differential diagnoses (Sibbald et al., 2021). It is therefore worth continuing this line of work into the mechanisms of this phenomenon, including its potential (de)biasing effect.

## Supplementary Information

**Additional file 1.** Supplementary Materials.

## Data Availability

The datasets used in the experiments are available at https://osf.io/zwqfh/.

## Footnotes

1 This expression originates from Einhorn and Hogarth’s “the persistence of the illusion of validity” (1978), whereby confidence is found to relate more to the number of prior judgements than the accuracy of those judgements.

2 ddx: the differential diagnoses list, the list of diagnostic alternatives that the software suggests.
