# Supplementary material for: Influences of early diagnostic suggestions on clinical reasoning
Source: Cogn Res Princ Implic. 2022 Dec 15;7:103. doi: 10.1186/s41235-022-00453-y (PMC9755454; doi:10.1186/s41235-022-00453-y)
Supplement: Supplementary file 1 — Additional file 1. Supplementary Materials. [file 41235_2022_453_MOESM1_ESM.docx]

**Supplementary Materials**

# Experiment 1

## S1. Patient scenarios and list of diagnostic suggestions

| **Scenario 1: Chest pain** |
| --- |
| **Patient name: James B.**  **Age:** 32 **BMI:** 24  **Smoking history:** Smoker (10 cigarettes a day).  **Past medical history:** Knee injury 2 years ago.    **Medications:** None.  **Last consultation:** Tonsillitis, 6 months ago.  **Presenting complaint:** *“Hi doctor, sorry to bother you but I've been getting this sharp pain in my chest for a few weeks now. I feel it every time that I take a breath. It seemed to start after a cold. I thought I’d better come and get it checked out.”* |
| **Additional neutral information** |
| **James also says:***"It's a sharp pain when I take a deep breath. I don’t feel it any other time. It’s only on the right side of my chest. I don't normally have a cough, but I have been coughing lately. It started with the cold. But I still have it 4 weeks later. I can't seem to shake it. It’s a dry cough, no phlegm or anything. I also feel a bit more tired than usual since all this started. Which is unusual for me, as I am normally quite a fit person, I cycle to work and like going to the gym regularly."*  **On examination**, his respiratory rate is 20 breaths per minute, and his pulse is 70. His temperature is 36.5 °C. His oxygen saturation is 98% on air, chest and heart examination are normal. No lymph nodes are felt. Pressing on the chest does not reproduce the pain. |

**Fig 1.** Chest pain scenario. Initially, most physicians were expected to consider musculoskeletal or pleuritic chest pain, as the patient is young and has no risk factors except for smoking. After the additional information, given the ongoing tiredness, most physicians were expected to think of other causes of the pain, such as acute or more chronic infection, pulmonary embolism, or pneumothorax. Also, the fact that James feels no pain when the physician presses his chest makes musculoskeletal pain less likely.

The list of diagnostic suggestions for James was the following:

- Acute LRTI
- Angina
- Anxiety Disorder
- Aortic valve regurgitation
- Aortic valve stenosis
- Asthma
- Cardiac arrhythmias
- Congestive cardiac failure
- Gastro-oesophageal reflux
- Herpes zoster
- Hyperthyroidism
- Lobar (pneumococcal) pneumonia
- Musculoskeletal chest pain
- Pneumothorax
- Pulmonary embolism
- Rib fracture
- Tuberculosis
- Type 2 diabetes mellitus

| **Scenario 2: Breathlessness** |
| --- |
| **Patient name: Derek H.**  **Age:**68 **BMI:**31  **Smoking history:** Smoker for 50 years. Currently smoking a packet-a-day but used to smoke 3 packets a day.  **Alcohol:** 12 units per week  **Past medical history:**Nothing of note.  **Medications:** None  **Last consultation:** Infected laceration of finger (in 2014)  **Appearance:** He appears comfortable at rest.  **Presenting complaint:** *"Well, it's my breathing doctor, that's the trouble. I get out of puff at the slightest thing. It's been coming on gradually for the past 6 months. The other day I was cutting the grass, and I came over all breathless with it. It went off, but to tell the truth, it did worry me a bit."* |
| **Additional neutral information** |
| **Derek also says:***"I get very tired these days. Yesterday, I sat down on the sofa to watch TV and dozed off for a few minutes.”*  **On examination**, his temperature is 36.7 °C, his blood pressure is 132/104, and his respiratory rate is 18 breaths per minute. There are no crackles to be heard in the chest. Examination of the abdomen reveals no abnormality. |

**Fig 2.** Breathlessness scenario. Initially, given his age and risk factors, most physicians were expected to consider chronic obstructive pulmonary disease (COPD) and/or ischaemic heart disease (IHD). Based on the additional information, COPD and IHD are still possible. However, the reported fatigue, along with his risk factors (i.e., smoking and obesity), introduce more diagnostic possibilities, such as heart failure and lung cancer.

The diagnostic suggestions for Derek were the following:

- Acute LRTI
- Allergic Reactions
- Angina
- Aortic valve regurgitation
- Aortic valve stenosis
- Asthma
- Atrial fibrillation
- Bronchitis
- Congestive cardiac failure
- COPD
- Cor Pulmonale
- Iron deficiency anaemias
- Lung Cancer
- Mitral stenosis
- Mitral valve regurgitation
- Multiple myeloma
- Myocardial infarction
- Non-Hodgkin's lymphoma
- Obstructive sleep apnoea
- Pulmonary Embolism
- Tonsillitis
- Tuberculosis
- URTI

Neither scenario contained sufficient information for a definite diagnosis. This was to capture physicians’ intuitive diagnostic hypotheses that come to mind first and also to limit the duration of the task. Providing full patient information would require more analytical processing and a longer response time.

## S2. Actively Open-minded Thinking Scale

1. Allowing oneself to be convinced by a solid opposing argument is a sign of good character.
2. People should take into consideration evidence that goes against conclusions they favour.
3. Being undecided or unsure is the result of muddled thinking (R).
4. People should revise their conclusions in response to relevant new information.
5. Changing your mind is a sign of weakness (R).
6. People should search actively for reasons why they might be wrong.
7. It is OK to ignore evidence against your established beliefs (R).
8. It is important to be loyal to your beliefs even when evidence is brought to bear against them (R).
9. When we are faced with a new question, the first answer that occurs to us is usually best (R).
10. Good thinking leads to uncertainty when there are good arguments on both sides.
11. When faced with a new question, we should consider more than one possible answer before reaching a conclusion.

For each statement, respondents are asked to indicate their agreement on a 5-point scale, ranging from “Completely disagree” to “Completely agree” with midpoint “Neutral”. (R) = reverse score.

## S3. Data classification

To make the data more easily analysable, Physicians’ responses were simplified according to BD’s suggestions by identifying synonyms or similar conditions and classifying them under a single category. Specifically in the chest pain scenario (James), Lower and Upper Respiratory Tract Infections were classified under “RTI” (Respiratory Tract Infection). Costochondritis and Musculoskeletal chest pain were classified under “MSK” (Musculoskeletal). Angina, Coronary Artery Disease, and Ischaemic Heart Disease (IHD) were classified under “Cardiac/Angina/IHD”. In the breathlessness scenario (Derek), carcinoma, malignancy and cancer were all classified under “Lung Cancer”.

To determine whether there was a change in diagnosis, we set the following two criteria:

1. In case of a single initial diagnosis, any change in diagnosis, including a switch to a different diagnosis or an addition of a new diagnosis, counted as a change.
2. In case of multiple initial diagnoses, any change in the diagnostic set, such as the addition or removal of diagnoses, was counted as a change.

## S4. Regression tables

| **Linear Regression (change in initial certainty)** | | | | | | | | |
| --- | --- | --- | --- | --- | --- | --- | --- | --- |
| **Step** | | **Estimate** | **Std. Error** | **df** | **t** | **Sig.** | **95% CI** | |
|  |  |  |  |  |  |  | **Lower Bound** | **Upper Bound** |
| 1 | Intercept | 1.315041 | 1.160713 | 209 | 1.133 | .259 | -.973147 | 3.603230 |
|  | Condition | -.238688 | .166799 | 194 | -1.431 | .154 | -.567657 | .090281 |
|  | AOT score | -.294500 | .267242 | 193 | -1.102 | .272 | -.821593 | .232594 |
|  | Certainty 1 | -.148767 | .054455 | 323 | -2.732 | .007 | -.255898 | -.041637 |
| 2 | Intercept | 1.217241 | 1.146194 | 208 | 1.062 | .289 | -1.042400 | 3.476882 |
|  | Condition | -.240694 | .166834 | 194 | -1.443 | .151 | -.569733 | .088345 |
|  | AOT score | -.231318 | .264807 | 192 | -.874 | .383 | -.753625 | .290989 |
|  | Certainty 1 | -.140119 | .054024 | 320 | -2.594 | .010 | -.246406 | -.033832 |
|  | Condition order | -.425126 | .170237 | 191 | -2.497 | .013 | -.760917 | -.089336 |

**Table 1.** Regression table of change in initial certainty

| **Logistic regression (change of diagnosis)** | | | | | | | | | | |
| --- | --- | --- | --- | --- | --- | --- | --- | --- | --- | --- |
| **Step** | | **Coefficient** | **Std. Error** | **t** | **Sig.** | **95% CI** | | **Exp(Coefficient)** | **95% CI for Exp(Coefficient)** | |
|  |  |  |  |  |  | **Lower** | **Upper** |  | **Lower** | **Upper** |
| 1 | Intercept | -.025 | 2.2094 | -.011 | .991 | -4.369 | 4.319 | .975 | .013 | 75.088 |
|  | Condition | .179 | .2074 | .862 | .389 | -.229 | .586 | 1.196 | .795 | 1.798 |
|  | AOT score | .292 | .3233 | .902 | .367 | -.344 | .928 | 1.339 | .709 | 2.528 |
|  | Certainty 1 | -.225 | .0687 | -3.275 | .001 | -.360 | -.090 | .799 | .698 | .914 |
| 2 | Intercept | .073 | 2.2124 | .033 | .974 | -4.277 | 4.423 | 1.076 | .014 | 83.371 |
|  | Condition | .184 | .2090 | .883 | .378 | -.226 | .595 | 1.203 | .797 | 1.814 |
|  | AOT score | .224 | .3279 | .684 | .494 | -.420 | .869 | 1.252 | .657 | 2.385 |
|  | Certainty 1 | -.237 | .0693 | -3.425 | .001 | -.373 | -.101 | .789 | .688 | .904 |
|  | Condition order | .493 | .2098 | 2.350 | .019 | .081 | .906 | 1.637 | 1.084 | 2.473 |

**Table 2.** Regression table of change of initial diagnosis

| **Poisson regression (number of investigations)** | | | | | | | | | | | | |
| --- | --- | --- | --- | --- | --- | --- | --- | --- | --- | --- | --- | --- |
| **Step** | | **B** | **Std. Error** | **95% Wald CI** | | **Hypothesis Test** | | | **Exp(B)** | **95% Wald CI for Exp(B)** | |  |
|  |  |  |  | **Lower** | **Upper** | **Wald Chi-Square** | **df** | **Sig.** |  | **Lower** | **Upper** |  |
| 1 | Intercept | -.446 | .3958 | -1.222 | .329 | 1.272 | 1 | .259 | .640 | .295 | 1.390 |  |
|  | Condition | -.007 | .0572 | -.119 | .106 | .014 | 1 | .907 | .993 | .888 | 1.111 |  |
|  | AOT score | .386 | .0898 | .210 | .562 | 18.445 | 1 | .000 | 1.471 | 1.233 | 1.753 |  |
|  | Certainty 1 | .007 | .0183 | -.029 | .043 | .134 | 1 | .714 | 1.007 | .971 | 1.044 |  |
| 2 | Intercept | -.488 | .3999 | -1.272 | .296 | 1.488 | 1 | .223 | .614 | .280 | 1.344 |  |
|  | Condition | -.006 | .0572 | -.119 | .106 | .013 | 1 | .910 | .994 | .888 | 1.111 |  |
|  | AOT score | .390 | .0898 | .214 | .566 | 18.831 | 1 | .000 | 1.477 | 1.238 | 1.761 |  |
|  | Certainty 1 | .007 | .0183 | -.028 | .043 | .165 | 1 | .685 | 1.007 | .972 | 1.044 |  |
|  | Condition order | .038 | .0574 | -.074 | .151 | .447 | 1 | .504 | 1.039 | .929 | 1.163 |  |

**Table 3.** Regression table of number of investigations ordered

# Experiment 2

## S5. Patient scenarios and list of diagnostic suggestions

| **Scenario 1: Chest pain** | |
| --- | --- |
| **Version 1: Cardiac chest pain (angina)** | **Version 2: Musculoskeletal chest pain** |
| **Patient name: David H.**  **Age:** 61 **BMI:** 27  **Smoking history**: Ex-smoker  **Past medical history:**Osteoarthritis of the knee (2016)  **Last consultation:** URTI a year ago.    **David says**: *"Hello doctor. I’ve been getting a pain in my chest for a week now. It gets worse when I work in my garden. Nothing else seems to affect it. I am a keen gardener, but I don't do any other exercise. I don't walk fast and avoid using stairs as I get knee pain. This pain in my chest usually goes away when I take a rest, doctor, but I thought it'd be good to come and see you."* | **Patient name: David H.**  **Age:** 51 **BMI:** 23  **Smoking history**: Never smoked  **Past medical history:**Osteoarthritis of the knee (2016)  **Last consultation:** URTI a year ago.    **David says**: *"Hello doctor. I’ve been getting a pain in my chest for a week now. I was helping my daughter move house and when I lifted the washing machine, I felt the pain coming on. I must have pulled a muscle in my chest, but my wife got a bit worried and asked me to come and see you."* |
| **Additional neutral information** | |
| - **Resting ECG results:** It is normal. - **Self-treatment:** David says that he has not taken any pain relief so far. He does not like taking tablets. - **General physical examination:** David's respiratory rate is 16 breaths per minute. His pulse is 80 beats per minute, regular and with a normal character. There is no wheeze in the chest and his heart sounds are normal. There is no peripheral oedema and the JVP is not raised. - **Family history of any significant illness:** There has not been any significant illness in David’s family, but his uncle had a myocardial infarction at age 70. - **Pain intensity:** David says that it is not really intense, but it does make him want to stop what he is doing. - **Chest pain during the night:** David says that it occasionally disturbs him at night. - **Other symptoms:** David does not report any other symptoms apart from the chest pain. | |

**Fig 3.** Version 1: Based on the initial description, most physicians were expected to think of a cardiac chest pain (i.e., angina), given the patient’s risk factors (age, smoking history, BMI) and the fact that the pain stops when resting. Version 2: Based on the initial description, physicians were expected to consider musculoskeletal chest pain as the most likely diagnosis, given that the patient has no risk factors and the pain started after a plausible chest injury (lifting a washing machine). Additional information: Based on this information, physicians were expected not to change their initial diagnosis, given that this information had minimal diagnostic value and did not help to differentiate between the possible diagnoses.

The list of diagnostic suggestions for scenario 1 was the following:

- Acute LRTI
- Angina
- Anxiety disorder
- Aortic valve regurgitation
- Aortic valve stenosis
- Asthma
- Gastro-oesophageal reflux
- Herpes zoster
- Musculoskeletal chest pain
- Pericarditis
- Pneumothorax
- Pulmonary Embolism

| **Scenario 2: Constipation** | |
| --- | --- |
| **Version 1: Colorectal cancer** | **Version 2: Irritable Bowel Syndrome** |
| **Patient name: Barbara H.**  **Age:** 61 **BMI:** 23.5  **Smoking history**: Never smoked  **Past medical history:**IBS diagnosis in 2015. Colonoscopy and coeliac screen normal.  **Last consultation:** Seven months ago, about loose stools, abdominal pain, and bloating. You ordered a full blood count. The results were:  Hb 11.2 g/dL* (12-15.5)  MCV 73 fL* (76-100) 10^9^  WCC 8.6 x 10^9^ (4.0-11.0)  Neutrophils 6 x 10^9^ (2.0-7.5)  Lymphocytes 2.1 x 10^9^ (1.3-3.5)  Monocytes 0.3 x 10^9^ (0.2-0.8)  Eosinophils 0.2 x 10^9^ (0-0.4)  Platelets 280 x 10^9^ (150-450)  Barbara was advised to get in touch about her results, but she did not.    **Barbara says**: *"Hello doctor. I'm having trouble with my bowels again. Not unusual for me, as you know. This time it's constipation. I've had hard stools for six weeks now, and it's very uncomfortable."* | **Patient name: Barbara H.**  **Age:** 61 **BMI:** 23.5  **Smoking history**: Never smoked  **Past medical history:**IBS diagnosis in 2015. Colonoscopy and coeliac screen normal.  **Last consultation:** Seven months ago, about loose stools, abdominal pain, and bloating. You ordered a full blood count. The results were:  Hb 13.6 g/dL (12-15.5)  MCV 88 fL (76-100) 10^9^  WCC 8.7 x 10^9^ (4.0-11.0)  Neutrophils 6.2 x 10^9^ (2.0-7.5)  Lymphocytes 2.8 x 10^9^ (1.3-3.5)  Monocytes 0.5 x 10^9^ (0.2-0.8)  Eosinophils 0.4 x 10^9^ (0-0.4)  Platelets 356 x 10^9^ (150-400)    **Barbara says**: *"Hello doctor. I'm having trouble with my bowels again. Not unusual for me, as you know. This time it's constipation. I've had hard stools for six weeks now, and it's very uncomfortable. Other than that, I feel OK. I don't feel tired or anything. Sometimes my tummy gets a bit painful, but it feels better after I've been to the loo."* |
| **Additional neutral information** | |
| - **Mucus in stools:** Barbara occasionally has mucus in her stools. - **Blood in stools:** Barbara has not had any blood in her stools. - **Sensation of bowel emptying:** Barbara reports that after she passed stools, she feels that she has emptied her bowel completely. - **Ferritin levels (current):** The result is 58μg/L (27-330). - **Digital rectal examination results:** The perianal region feels normal. The sphincter tone is normal. You feel no lumps, blood or faeces. - **Previous episodes of bowel problems:** Barbara occasionally experiences episodes of either loose or hard stools, which may last for a few weeks. Her last bout of bowel trouble was seven months ago, when she had loose stools for a while. - **Family history of any significant illness:** There is no family history of any significant illness. | |

**Fig 4.** Version 1: At this stage, physicians were expected to consider colorectal cancer as the most likely diagnosis for Barbara, given her low level of haemoglobin. Version 2: Given this initial description, physicians were expected to consider IBS as the most likely diagnosis. Additional information: Based on this information, physicians were expected not to change their initial diagnosis.

The list of diagnostic suggestions for scenario 1 was the following:

- Anxiety disorder
- Coeliac disease
- Colorectal cancer
- Diverticular disease
- Gastroenteritis
- Inflammatory bowel disease
- Irritable bowel syndrome
- Malignant neoplasm of pancreas
- Malignant neoplasm of stomach
- Ovarian cancer
- Type 2 diabetes mellitus
- Uterine leiomyoma – fibroids

## S6. Data classification

Physicians’ diagnoses we simplified and organised into categories according to BD’s suggestions. In the chest pain scenario (David), diagnoses related to cardiac causes (e.g., angina, pericarditis, ischaemic heart disease) were classified under “cardiac”. Likewise, diagnoses related to musculoskeletal causes (e.g., costochondritis, musculoskeletal chest pain, pulled muscle) were classified under “MSK”. In the constipation scenario (Barbara), diagnoses related to cancer (e.g., colon cancer, bowel cancer, lower gastrointestinal malignancy) were classified under “cancer”. IBS diagnosis stood on its own as a category. Likewise, the rest of the diagnoses were not classified into a category.

We also measured changes in diagnoses within each patient scenario. If physicians’ initial diagnosis was different from their final diagnosis, then this would count as a diagnostic change. In addition to this criterion, if physicians provided a single initial diagnosis and multiple final diagnoses, then this would also count as a change.

## S7. Regression tables

| **Poisson regressions (number of cues requested)** | | | | | | | | | | |
| --- | --- | --- | --- | --- | --- | --- | --- | --- | --- | --- |
| **Step** | | **Coefficient** | **Std. Error** | **t** | **Sig.** | **95% Cl** | | **Exp(Coefficient)** | **95% CI for Exp(Coefficient)** | |
|  |  |  |  |  |  | **Lower** | **Upper** |  | **Lower** | **Upper** |
| 1 | Intercept | .721 | .2371 | 3.043 | .002 | .256 | 1.187 | 2.057 | 1.291 | 3.278 |
|  | Condition | -.052 | .0434 | -1.189 | .235 | -.137 | .034 | .950 | .872 | 1.034 |
|  | AOT score | .315 | .0567 | 5.551 | .000 | .203 | .426 | 1.370 | 1.225 | 1.531 |
|  | Certainty 1 | -.085 | .0120 | -7.098 | .000 | -.109 | -.062 | .918 | .897 | .940 |
|  | Severity | .054 | .0447 | 1.207 | .228 | -.034 | .142 | 1.055 | .967 | 1.152 |

**Table 4.** Regression table of cue requests.

| **Linear regression (perceived cue support)** | | | | | | | | |
| --- | --- | --- | --- | --- | --- | --- | --- | --- |
| **Step** | | **Estimate** | **Std. Error** | **df** | **t** | **Sig.** | **95% CI** | |
|  |  |  |  |  |  |  | **Lower Bound** | **Upper Bound** |
| 1 | Intercept | 3.166415 | 1.009299 | 294 | 3.137 | .002 | 1.180059 | 5.152772 |
|  | Condition | .366869 | .147215 | 244 | 2.492 | .013 | .076893 | .656845 |
|  | AOT score | .040986 | .239129 | 253 | .171 | .864 | -.429954 | .511926 |
|  | Certainty 1 | .516915 | .048512 | 490 | 10.656 | .000 | .421599 | .612231 |
|  | Severity | -2.05153 | .171668 | 468 | -11.951 | .000 | -2.388864 | -1.714196 |

**Table 5.** Regression table of perceived cue support.

| **Linear regression (change in initial certainty)** | | | | | | | | | | | | | | | | |
| --- | --- | --- | --- | --- | --- | --- | --- | --- | --- | --- | --- | --- | --- | --- | --- | --- |
| **Step** | | | **Estimate** | | **Std. Error** | | **df** | | **t** | | **Sig.** | | **95% CI** | | | |
|  |  |  |  |  |  |  |  |  |  |  |  |  | **Lower Bound** | | **Upper Bound** | |
| 1 | Intercept | 2.651949 | | .867036 | | 296 | | 3.059 | | .002 | | .945623 | | 4.358274 | |  |
|  | Condition | .033924 | | .156615 | | 245 | | .217 | | .829 | | -.274558 | | .342407 | |  |
|  | AOT score | .100715 | | .201698 | | 254 | | .499 | | .618 | | -.296495 | | .497926 | |  |
|  | Certainty 1 | -.318214 | | .045388 | | 471 | | -7.011 | | .000 | | -.407402 | | -.229026 | |  |
|  | Severity | -1.41327 | | .164276 | | 491 | | -8.603 | | .000 | | -1.736046 | | -1.090506 | |  |

**Table 6.** Regression table of change in initial certainty.

| **Logistic regression (change of initial diagnosis)** | | | | | | | | | | |
| --- | --- | --- | --- | --- | --- | --- | --- | --- | --- | --- |
| **Step** | | **Coefficient** | **Std. Error** | **t** | **Sig.** | **95% CI** | | **Exp(Coefficient)** | **95% CI for Exp(Coefficient)** | |
|  |  |  |  |  |  | **Lower** | **Upper** |  | **Lower** | **Upper** |
| 1 | Intercept | .181 | 1.8261 | .099 | .921 | -3.407 | 3.769 | 1.198 | .033 | 43.318 |
|  | Condition | -.203 | .2470 | -.824 | .411 | -.689 | .282 | .816 | .502 | 1.326 |
|  | AOT score | .028 | .3119 | .090 | .929 | -.585 | .641 | 1.028 | .557 | 1.898 |
|  | Certainty 1 | -.385 | .0701 | -5.497 | .000 | -.523 | -.248 | .680 | .593 | .781 |
|  | Severity | .918 | .2607 | 3.520 | .000 | .405 | 1.430 | 2.504 | 1.500 | 4.179 |

**Table 7.** Regression table of change in diagnosis.

| **Poisson regression (number of differential diagnoses)** | | | | | | | | | | |
| --- | --- | --- | --- | --- | --- | --- | --- | --- | --- | --- |
| **Step** | | **Coefficient** | **Std. Error** | **t** | **Sig.** | **95% CI** | | **Exp(Coefficient)** | **95% CI for Exp(Coefficient)** | |
|  |  |  |  |  |  | **Lower** | **Upper** |  | **Lower** | **Upper** |
| 1 | Intercept | -.079 | .4115 | -.191 | .848 | -.887 | .730 | .924 | .412 | 2.075 |
|  | Condition | .060 | .0761 | .789 | .430 | -.089 | .210 | 1.062 | .914 | 1.233 |
|  | AOT score | .192 | .0976 | 1.969 | .049 | .000 | .384 | 1.212 | 1.000 | 1.468 |
|  | Certainty 1 | -.050 | .0212 | -2.370 | .018 | -.092 | -.009 | .951 | .912 | .991 |
|  | Severity | -.140 | .0784 | -1.788 | .074 | -.294 | .014 | .869 | .745 | 1.014 |

**Table 8.** Regression table of differential diagnoses.

# Experiment 3

## S8. Regression tables

| **Poisson regression of number of cues requested (Control vs. Aided 2)** | | | | | | | | | | | |
| --- | --- | --- | --- | --- | --- | --- | --- | --- | --- | --- | --- |
| **Step** | | **B** | **Std. Error** | **95% Wald CI** | | **Hypothesis Test** | | | **Exp(B)** | **95% Wald CI for Exp(B)** | |
|  |  |  |  | **Lower** | **Upper** | **Wald χ^2^** | **df** | **Sig.** |  | **Lower** | **Upper** |
| 1 | Intercept | 1.389 | .2827 | .835 | 1.943 | 24.160 | 1 | .000 | 4.013 | 2.306 | 6.983 |
|  | Condition | -.042 | .0492 | -.139 | .054 | .742 | 1 | .389 | .959 | .870 | 1.056 |
|  | AOT score | .173 | .0657 | .044 | .302 | 6.949 | 1 | .008 | 1.189 | 1.045 | 1.353 |
|  | Certainty 1 | -.099 | .0133 | -.125 | -.073 | 55.292 | 1 | .000 | .906 | .882 | .930 |
|  | Severity | -.003 | .0499 | -.101 | .095 | .003 | 1 | .957 | .997 | .904 | 1.100 |

**Table 9.** Regression table of number of cues requested (Control vs. Aided 2).

| **Poisson regression of number of cues requested (Aided 1 vs. Aided 2)** | | | | | | | | | | | |
| --- | --- | --- | --- | --- | --- | --- | --- | --- | --- | --- | --- |
| **Step** | | **B** | **Std. Error** | **95% Wald CI** | | **Hypothesis Test** | | | **Exp(B)** | **95% Wald CI for Exp(B)** | |
|  |  |  |  | **Lower** | **Upper** | **Wald χ^2^** | **df** | **Sig.** |  | **Lower** | **Upper** |
| 1 | Intercept | 1.574 | .2917 | 1.003 | 2.146 | 29.130 | 1 | .000 | 4.827 | 2.725 | 8.549 |
|  | Condition | .015 | .0490 | -.081 | .111 | .092 | 1 | .762 | 1.015 | .922 | 1.117 |
|  | AOT score | .107 | .0678 | -.026 | .240 | 2.485 | 1 | .115 | 1.113 | .974 | 1.271 |
|  | Certainty 1 | -.089 | .0140 | -.117 | -.062 | 40.819 | 1 | .000 | .915 | .890 | .940 |
|  | Severity | .042 | .0494 | -.055 | .138 | .707 | 1 | .400 | 1.042 | .946 | 1.148 |

**Table 10.** Regression table of number of cues requested (Aided 1 vs. Aided 2).

| **Linear regression of perceived cue support (Control vs. Aided 2)** | | | | | | | | |
| --- | --- | --- | --- | --- | --- | --- | --- | --- |
| **Step** | | **Unstandardized Coefficients** | | **Standardized Coefficients** | **t** | **Sig.** | **95.0% CI for B** | |
|  |  | **B** | **Std. Error** | **Beta** |  |  | **Lower Bound** | **Upper Bound** |
| 1 | (Constant) | 3.181 | 1.190 |  | 2.672 | .008 | .841 | 5.521 |
|  | Condition | .100 | .104 | .040 | .965 | .335 | -.104 | .305 |
|  | AOT score | .189 | .271 | .029 | .699 | .485 | -.343 | .722 |
|  | Certainty 1 | .461 | .060 | .326 | 7.634 | .000 | .342 | .579 |
|  | Severity | -2.709 | .212 | -.543 | -12.751 | .000 | -3.127 | -2.291 |

**Table 11.** Regression model of perceived cue support (Control vs. Aided 2).

| **Linear regression of perceived cue support (Aided 1 vs. Aided 2)** | | | | | | | | |
| --- | --- | --- | --- | --- | --- | --- | --- | --- |
| **Step** | | **Unstandardized Coefficients** | | **Standardized Coefficients** | **t** | **Sig.** | **95.0% CI for B** | |
|  |  | **B** | **Std. Error** | **Beta** |  |  | **Lower Bound** | **Upper Bound** |
| 1 | (Constant) | 2.731 | 1.141 |  | 2.393 | .017 | .487 | 4.975 |
|  | Condition | -.230 | .190 | -.052 | -1.213 | .226 | -.604 | .143 |
|  | AOT score | .403 | .255 | .068 | 1.578 | .115 | -.099 | .904 |
|  | Certainty 1 | .452 | .056 | .352 | 8.015 | .000 | .341 | .562 |
|  | Severity | -2.074 | .192 | -.471 | -10.791 | .000 | -2.452 | -1.696 |

**Table 12.** Regression model of perceived cue support (Aided 1 vs. Aided 2).

| **Linear Regression of change in initial certainty (Control vs. Aided 2)** | | | | | | | | |
| --- | --- | --- | --- | --- | --- | --- | --- | --- |
| **Step** | | **Unstandardized Coefficients** | | **Standardized Coefficients** | **t** | **Sig.** | **95.0% CI for B** | |
|  |  | **B** | **Std. Error** | **Beta** |  |  | **Lower Bound** | **Upper Bound** |
| 1 | (Constant) | 2.303 | 1.090 |  | 2.113 | .035 | .160 | 4.447 |
|  | Condition | -.013 | .095 | -.006 | -.140 | .889 | -.201 | .174 |
|  | AOT score | .139 | .248 | .025 | .562 | .574 | -.348 | .627 |
|  | Certainty 1 | -.258 | .055 | -.209 | -4.666 | .000 | -.367 | -.149 |
|  | Severity | -1.868 | .195 | -.429 | -9.599 | .000 | -2.250 | -1.485 |

**Table 13.** Regression table of change in initial certainty (Control vs. Aided 2).

| **Linear Regression of change in initial certainty (Aided 1 vs. Aided 2)** | | | | | | | | |
| --- | --- | --- | --- | --- | --- | --- | --- | --- |
| **Step** | | **Unstandardized Coefficients** | | **Standardized Coefficients** | **t** | **Sig.** | **95.0% CI for B** | |
|  |  | **B** | **Std. Error** | **Beta** |  |  | **Lower Bound** | **Upper Bound** |
| 1 | (Constant) | 2.436 | 1.109 |  | 2.195 | .029 | .254 | 4.617 |
|  | Condition | -.288 | .185 | -.071 | -1.561 | .119 | -.651 | .075 |
|  | AOT score | .279 | .248 | .052 | 1.123 | .262 | -.209 | .766 |
|  | Certainty 1 | -.326 | .055 | -.276 | -5.952 | .000 | -.434 | -.218 |
|  | Severity | -1.309 | .187 | -.324 | -7.009 | .000 | -1.677 | -.942 |

**Table 14.** Regression table of change in initial certainty (Aided 1 vs. Aided 2).

| **Logistic regression of diagnostic change (Control vs. Aided 2)** | | | | | | | | | |
| --- | --- | --- | --- | --- | --- | --- | --- | --- | --- |
| **Step** | | **B** | **S.E.** | **Wald** | **df** | **Sig.** | **Exp(B)** | **95% CI.for Exp(B)** | |
|  |  |  |  |  |  |  |  | **Lower** | **Upper** |
| 1 | Condition | -.041 | .141 | .086 | 1 | .769 | .960 | .728 | 1.264 |
|  | AOT score | -.010 | .365 | .001 | 1 | .979 | .990 | .484 | 2.027 |
|  | Certainty 1 | -.473 | .082 | 33.373 | 1 | .000 | .623 | .531 | .732 |
|  | Severity | 1.022 | .297 | 11.878 | 1 | .001 | 2.779 | 1.554 | 4.969 |
|  | Constant | .815 | 1.570 | .270 | 1 | .604 | 2.259 |  |  |

**Table 15.** Regression table of change of initial diagnosis (Control vs. Aided 2).

| **Logistic regression of diagnostic change (Aided 1 vs. Aided 2)** | | | | | | | | | |
| --- | --- | --- | --- | --- | --- | --- | --- | --- | --- |
| **Step** | | **B** | **S.E.** | **Wald** | **df** | **Sig.** | **Exp(B)** | **95% CI.for Exp(B)** | |
|  |  |  |  |  |  |  |  | **Lower** | **Upper** |
| 1 | Condition | .762 | .322 | 5.596 | 1 | .018 | 2.143 | 1.140 | 4.030 |
|  | AOT score | -.405 | .437 | .859 | 1 | .354 | .667 | .283 | 1.571 |
|  | Certainty 1 | -.458 | .092 | 25.071 | 1 | .000 | .632 | .528 | .757 |
|  | Severity | .546 | .318 | 2.959 | 1 | .085 | 1.727 | .927 | 3.218 |
|  | Constant | .969 | 1.922 | .254 | 1 | .614 | 2.636 |  |  |

**Table 16.** Regression table of change of initial diagnosis (Aided 1 vs. Aided 2).

| **Poisson regression of number of differential diagnoses (Control vs. Aided 2)** | | | | | | | | | | | |
| --- | --- | --- | --- | --- | --- | --- | --- | --- | --- | --- | --- |
| **Step** | | **B** | **Std. Error** | **95% Wald CI** | | **Hypothesis Test** | | | **Exp(B)** | **95% Wald CI for Exp(B)** | |
|  |  |  |  | **Lower** | **Upper** | **Wald χ^2^** | **df** | **Sig.** |  | **Lower** | **Upper** |
| 1 | Intercept | -.540 | .5258 | -1.570 | .491 | 1.054 | 1 | .305 | .583 | .208 | 1.633 |
|  | Condition | -.032 | .0893 | -.207 | .143 | .127 | 1 | .721 | .969 | .813 | 1.154 |
|  | AOT score | .340 | .1213 | .102 | .578 | 7.855 | 1 | .005 | 1.405 | 1.108 | 1.782 |
|  | Certainty 1 | -.078 | .0245 | -.126 | -.030 | 10.128 | 1 | .001 | .925 | .881 | .970 |
|  | Severity | -.114 | .0907 | -.292 | .064 | 1.584 | 1 | .208 | .892 | .747 | 1.066 |

**Table 17.** Regression table of the number of differential diagnoses (Control vs. Aided 2).

| **Poisson regression of number of differential diagnoses (Aided 1 vs. Aided 2)** | | | | | | | | | | | |
| --- | --- | --- | --- | --- | --- | --- | --- | --- | --- | --- | --- |
| **Step** | | **B** | **Std. Error** | **95% Wald CI** | | **Hypothesis Test** | | | **Exp(B)** | **95% Wald CI for Exp(B)** | |
|  |  |  |  | **Lower** | **Upper** | **Wald χ^2^** | **df** | **Sig.** |  | **Lower** | **Upper** |
| 1 | Intercept | .162 | .5498 | -.916 | 1.239 | .087 | 1 | .769 | 1.176 | .400 | 3.453 |
|  | Condition | -.079 | .0915 | -.258 | .101 | .741 | 1 | .389 | .924 | .773 | 1.106 |
|  | AOT score | .205 | .1280 | -.045 | .456 | 2.576 | 1 | .108 | 1.228 | .956 | 1.578 |
|  | Certainty 1 | -.098 | .0257 | -.148 | -.048 | 14.535 | 1 | .000 | .907 | .862 | .953 |
|  | Severity | -.217 | .0924 | -.398 | -.036 | 5.536 | 1 | .019 | .805 | .671 | .964 |

**Table 18.** Regression table of the number of differential diagnoses (Aided 1 vs. Aided 2).

## S9. Means of perceived cue support across scenario versions

| Clinical cue | Control  Mean (SD) | Aided 1  Mean (SD) | Aided 2  Mean (SD) | Overall rating Mean (SD) |
| --- | --- | --- | --- | --- |
| General physical examination | 6.19 (2.10) | 6.92 (1.70) | 5.23 (2.58) | 6.23 (2.17) |
| Family history | 5.47 (2.68) | 5.82 (2.15) | 5.27 (1.98) | 5.53 (2.30) |
| Pain intensity | 4.96 (2.35) | 6.11 (2.09) | 5.14 (2.07) | 5.47 (2.21) |
| Self-treatment | 4.66 (3.04) | 5.67 (2.61) | 6.02 (2.68) | 5.53 (2.81) |
| Chest pain during the night | 5.86 (2.76) | 5.33 (2.07) | 6.24 (2.38) | 5.82 (2.42) |
| Resting ECG results | 6.84 (2.14) | 6.69 (1.70) | 5.32 (2.81) | 6.36 (2.30) |
| Other symptoms | 6.80 (2.10) | 6.69 (1.87) | 6.89 (1.36) | 6.77 (1.93) |
| Overall | **6.32 (1.97)** | **6.49 (1.51)** | **6.28 (1.83)** | **6.36 (1.78)** |

**Table 19.** Mean cue ratings in chest pain scenario, Musculoskeletal version.

| Clinical cue | Control  Mean (SD) | Aided 1  Mean (SD) | Aided 2  Mean (SD) | Overall rating Mean (SD) |
| --- | --- | --- | --- | --- |
| General physical examination | 4.30 (2.53) | 6.14 (2.21) | 5.36 (2.49) | 5.27 (2.51) |
| Family history | 5.72 (2.75) | 6.35 (1.74) | 6.16 (1.95) | 5.66 (2.35) |
| Pain intensity | 6.05 (2.82) | 6.73 (1.40) | 4.70 (2.64) | 5.80 (2.47) |
| Self-treatment | 2.94 (2.17) | 3.93 (2.40) | 5.43 (2.65) | 4.62 (2.69) |
| Chest pain during the night | 4.29 (2.79) | 5.14 (1.82) | 4.56 (2.73) | 4.61 (2.57) |
| Resting ECG results | 4.05 (2.86) | 5.61 (2.47) | 3.59 (2.78) | 4.60 (2.80) |
| Other symptoms | 3.95 (2.85) | 6.32 (1.95) | 5.50 (2.37) | 5.21 (2.64) |
| Overall | **4.94 (2.55)** | **6.01 (1.70)** | **5.25 (2.10)** | **5.40 (2.19)** |

**Table 20.** Mean cue ratings in chest pain scenario, cardiac chest pain (angina) version.

| Clinical cue | Control  Mean (SD) | Aided 1  Mean (SD) | Aided 2  Mean (SD) | Overall rating Mean (SD) |  |
| --- | --- | --- | --- | --- | --- |
| Digital rectal examination | 7.53 (1.38) | 6.89 (1.56) | 7.41 (1.82) | 7.25 (1.57) |  |
| Family history | 6.52 (2.58) | 6.21 (2.42) | 6.83 (2.04) | 6.40 (2.42) |  |
| Mucus in stools | 5.94 (2.46) | 5.50 (2.55) | 7.24 (2.05) | 6.37 (2.42) |  |
| Blood in stools | 7.51 (1.45) | 6.71 (1.35) | 7.23 (1.87) | 7.14 (1.54) |  |
| Ferritin levels (current) | 7.73 (1.15) | 7.00 (1.68) | 7.42 (1.91) | 7.36 (1.64) |  |
| Previous episodes of bowel problems | 7.91 (1.63) | 6.24 (1.82) | 7.38 (1.80) | 7.25 (1.85) |  |
| Sensation of bowel emptying | 7.28 (2.92) | 6.83 (1.43) | 7.61 (1.87) | 7.26 (1.70) |  |
| Overall | **7.29 (1.36)** | **6.74 (1.48)** | **7.36 (1.38)** | **7.13 (1.43)** | |

**Table 21.** Mean cue ratings in constipation scenario, IBS version.

| Clinical cue | Control  Mean (SD) | Aided 1  Mean (SD) | Aided 2  Mean (SD) | Overall rating Mean (SD) |  |
| --- | --- | --- | --- | --- | --- |
| Digital rectal examination | 2.79 (2.31) | 3.93 (2.73) | 4.36 (3.37) | 3.58 (2.81) |  |
| Family history | 1.83 (1.82) | 3.75 (2.52) | 0.67 (1.15) | 2.68 (2.39) |  |
| Mucus in stools | 3.58 (2.15) | 4.07 (1.68) | 3.10 (2.15) | 3.44 (2.05) |  |
| Blood in stools | 3.28 (2.74) | 3.85 (2.26) | 3.19 (3.04) | 3.49 (2.62) |  |
| Ferritin levels (current) | 1.92 (2.43) | 3.14 (2.21) | 3.19 (2.53) | 2.79 (2.44) |  |
| Previous episodes of bowel problems | 3.71 (2.56) | 4.60 (2.23) | 3.05 (2.35) | 3.77 (2.44) |  |
| Sensation of bowel emptying | 3.00 (2.55) | 3.40 (2.72) | 3.43 (2.50) | 3.29 (2.55) |  |
| Overall | **3.16 (2.57)** | **3.99 (2.26)** | **3.16 (2.28)** | **3.44 (2.39)** | |

**Table 22.** Mean cue ratings in constipation scenario, colorectal cancer version.
